# Limited haplotype diversity underlies polygenic trait architecture across 70 years of wheat breeding

**DOI:** 10.1186/s13059-021-02354-7

**Published:** 2021-05-06

**Authors:** Michael F. Scott, Nick Fradgley, Alison R. Bentley, Thomas Brabbs, Fiona Corke, Keith A. Gardner, Richard Horsnell, Phil Howell, Olufunmilayo Ladejobi, Ian J. Mackay, Richard Mott, James Cockram

**Affiliations:** 1 University College London (UCL) Genetics Institute, Gower St, London, WC1E 6BT UK; 2 Current address: School of Biological Sciences, University of East Anglia, Norwich Research Park, Norwich, NR4 7TJ UK; 3 National Institute for Agricultural Botany (NIAB), 93 Lawrence Weaver Road, Cambridge, CB3 0LE UK; 4 Current address: International Maize and Wheat Improvement Center (CIMMYT), El Batán, Texcoco, Mexico; 5 Earlham Institute, Norwich, NR4 7UZ UK; 6 The National Plant Phenomics Centre, Institute of Biological, Rural and Environmental Sciences (IBERS), Aberystwyth University, Gogerddan, Aberystwyth, SY23 3EE UK; 7 Current address: SRUC, Peter Wilson Building King’s Buildings, W Mains Rd, Edinburgh, EH9 3JG UK

**Keywords:** Wheat, MAGIC, Multi-parent population, Imputation, Low-coverage whole-genome sequencing, Genomic prediction, GWAS, Phenomics, Pleiotropy

## Abstract

**Background:**

Selection has dramatically shaped genetic and phenotypic variation in bread wheat. We can assess the genomic basis of historical phenotypic changes, and the potential for future improvement, using experimental populations that attempt to undo selection through the randomizing effects of recombination.

**Results:**

We bred the NIAB Diverse MAGIC multi-parent population comprising over 500 recombinant inbred lines, descended from sixteen historical UK bread wheat varieties released between 1935 and 2004. We sequence the founders’ genes and promoters by capture, and the MAGIC population by low-coverage whole-genome sequencing. We impute 1.1 M high-quality SNPs that are over 99% concordant with array genotypes. Imputation accuracy only marginally improves when including the founders’ genomes as a haplotype reference panel. Despite capturing 73% of global wheat genetic polymorphism, 83% of genes cluster into no more than three haplotypes. We phenotype 47 agronomic traits over 2 years and map 136 genome-wide significant associations, concentrated at 42 genetic loci with large and often pleiotropic effects. Around half of these overlap known quantitative trait loci. Most traits exhibit extensive polygenicity, as revealed by multi-locus shrinkage modelling.

**Conclusions:**

Our results are consistent with a gene pool of low haplotypic diversity, containing few novel loci of large effect. Most past, and projected future, phenotypic changes arising from existing variation involve fine-scale shuffling of a few haplotypes to recombine dozens of polygenic alleles of small effect. Moreover, extensive pleiotropy means selection on one trait will have unintended consequences, exemplified by the negative trade-off between yield and protein content, unless selection and recombination can break unfavorable trait-trait associations.

**Supplementary Information:**

The online version contains supplementary material available at 10.1186/s13059-021-02354-7.

## Introduction

Bread wheat (*Triticum aestivum* L.) production is a critical component of worldwide food security. About 21% of the calories and protein consumed by humans are from wheat, and demand is predicted to increase 60% between 2014 and 2050 [1], by which time the human population will have reached 9 billion. With genetic gains in yield currently ~ 1% per year [2], we need a genetic and genomic toolbox to sustain wheat improvement. These include a high-quality annotated reference assembly for the 17-Gb hexaploid bread wheat genome [3] and surveys of standing genetic variation. Global wheat variation surveys are available from, for example, resequencing and de novo assembly of 15 accessions [4], whole-genome resequencing (WGS) of 93 accessions [5], genotyping by sequencing (~ 16 k markers) for ~ 17 k breeding program lines [6], and genotyping array data for collections of 804 [7] and 4500 [8] accessions (~ 15 k and ~ 113 k markers, respectively). Such genomic datasets are associated with varying levels of phenotypic information, e.g., five traits measured in 2 years for 870 global accessions with exome capture data [9] and 12 traits measured in 2 years, six locations, and three cropping intensities for 191 German varieties with genotyping array data (~ 9 k markers) [10]. When available, such data reveal genotype-phenotype associations and thereby aid genetic gain through breeding [6].

Rather than examining existing varieties or breeding lines, in which genotype-trait and trait-trait associations may be confounded by population structure or hidden by low allele frequencies, we constructed an experimental population using 16 inbred founders through hundreds of structured intercrosses. The founders, chosen to maximize the genetic diversity captured in a historical winter wheat panel, were released in the UK between 1935 and 2004 (Additional file 1: Table S1) and crossed together to create a Multiparent Advanced Generation Intercross (MAGIC) population (termed “NIAB DIVERSE MAGIC,” hereafter “NDM”) of over 500 recombinant inbred lines (RILs). Compared to other crossing designs, MAGIC populations accumulate more recombination events, thereby increasing mapping resolution while simultaneously capturing high levels of genetic and phenotypic variation with little population structure [11, 12]. Because MAGIC populations are genetically diverse while eliminating low-frequency variation, they make powerful, general purpose tools for dissecting trait genetic architectures in the wider germplasm [13].

NDM differs from other wheat MAGIC populations [14–17] by capturing and re-shuffling the genomes of 16 historical wheats, rather than a smaller number of modern elite varieties. The 16 founders were intercrossed over four generations in 15 funnels. Each funnel was initiated from a non-overlapping subset of eight of the 120 independent F1 combinations as described in [18]. The use of a larger number of founders, selected on historical diversity and intercrossed in multiple funnels, creates a greater number and more uniform genome-wide distribution of recombinant haplotypes than commonly used alternative multi-parent populations [18].

We characterized NDM genetic variation using promoter-gene capture [19] for the 16 founders and low-coverage whole-genome sequencing (WGS) at ~ 0.3× of the > 500 RILs, from which we were able to impute accurate RIL genotypes. We measured 47 phenotypes, of which 25 were assessed across two growing seasons. We make the NDM germplasm, genotypic and phenotypic resources publicly accessible, serving as a stable and generic resource for trait mapping and prediction.

We asked the following questions. First, what genetic variation segregates among the MAGIC founders, how does it reflect global wheat diversity, and specifically how many distinct haplotypes typically segregate at each locus? Second, how does this variation underpin agronomic traits, as revealed through genetic mapping and genomic prediction? And third, how can knowledge of pleiotropic genetic control of trait relationships be used to define selection strategies to optimize trade-offs among traits for achieving further genetic gain?

## Results

### NIAB Diverse MAGIC founders

The 16 founders were selected from a panel of 94 historical varieties released in the UK over a ~ 70-year period (Additional file 1: Table S1), using 546 Diversity Array Technology (DArT) and 61 single sequence repeat (SSR) markers [20]. We sequenced 15 founders after enrichment using capture probe sets [19] for both genic regions and putative promoters at average coverage of 22.94× of the targets (Additional file 1: Table S1). The remaining founder, Holdfast, was sequenced by whole genome sequencing (WGS), but to ensure consistency across founders, we restricted our attention to the capture targets, at which coverage in Holdfast was 15.8×. We sequenced using Illumina 150 bp paired end reads whose combined span often included sequence differences between homeologous loci on the A, B, and D subgenomes of hexaploid wheat, thereby resolving many otherwise ambiguous alignments. Furthermore, we only used high-quality alignments (mapQ> 30) for coverage calculations and variant calling, and excluded variant sites with missing or heterozygous calls in any founder (e.g., from homeologous variation and misalignment). After quality control, we called 1.13 M high-quality single-nucleotide polymorphisms (SNPs) across the 110,790 promoter-gene pairs targeted by the capture probes [19] spanning 557 Mb in total, (summarized in Additional file 2: Figure S1). Only 97,727 SNPs (8.7%) were on the D subgenome and almost half (17,289/35,021, 49.4%) of the promoter-gene pairs on the D subgenome had no SNPs passing quality control, compared to 26.6% (9656/36,302) and 21.7% (8012/36,738) on the A and B subgenomes, respectively. A comparative lack of diversity is expected on the D subgenome as it was acquired in the most recent allo-polyploidization event.

We characterized the functional impact of the 1.13 M SNPs called in the founders using Variant Effect Predictor software v2.0 [21]. This method estimates the location and impact of variants using gene annotations. In total, 189,459 SNPs (16.9%) were in exons, 44,268 (3.9%) were in untranslated regions (UTRs), 294,019 (26.2%) were in introns, and 565,119 (50.4%) were upstream or downstream of genes. The remaining 2.6% fell in splice regions or multiple categories. Of exonic SNPs, 94,998 (50.1%) were “missense” variants that affect amino acid sequence and 92,215 (48.7%) were “synonymous” variants that do not change the amino acid sequence. The remaining exonic SNPs affected stop codons or non-coding exon transcripts. In short, the majority of called SNPs (91%) do not directly affect protein sequence, and we were unable to find SNPs that were strong functional candidates for the quantitative trait loci (QTLs) identified below. These results are consistent with recent wheat genome assemblies, which suggest that gene-flanking sequences are required to distinguish varieties [22]. For this reason, the use of promoter capture or complete de novo assembly may significantly enhance the ability of called SNPs to tag important functional variation.

**Fig. 1:**
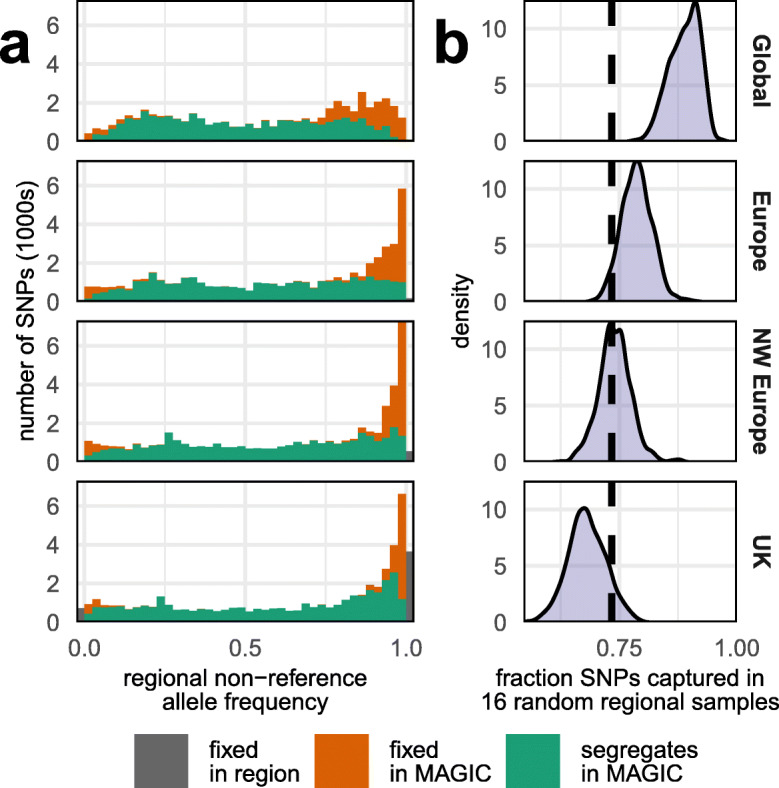
The NDM population is representative of NW European wheat. **a** SNPs segregating (green) or fixed (orange) in NDM at 50,335 sites in 4506 global wheats, grouped into “Global,” “European,” “North-West European,” and “UK” nested subpopulations and binned by the allele frequency in these subpopulations. **b** The fraction of sites that are polymorphic in 16 randomly chosen wheats from each subpopulation based on 1000 random replications. The dashed vertical black line at *x* = 0.734 is the fraction of SNPs segregating among NDM founders

We placed the 16 founders in the context of global wheat diversity by analyzing 113,457 genotyping array sites that vary among 4506 diverse global wheat accessions [8], of which 50,335 sites were callable across all founders. We classified global wheats into nested subsets representing the UK only (*n* = 154), North-West (NW) Europe (*n* = 1343), Europe (*n* = 2331), and Global (*n* = 4506), to understand how allele frequencies across subsets relate to our founders (Fig. 1). Most Global common variants are polymorphic in the founders whereas rare alleles are more likely to be fixed. Of 10,111 genotyping array SNPs that are rare in the Global germplasm, which we defined to have minor allele frequencies (MAF) below 0.05, 4873 (48%) segregate among the 16 founders, (Additional file 2: Figure S2). We next asked whether we could have selected 16 founders that sampled the variation space more comprehensively. We simulated selections from the same nested subsets and compared the distribution of the fraction of segregating sites with that in the actual NDM founders, and found the latter capture more diversity than an average selection of UK wheats, about average diversity for NW European wheats, but less than average for wider European and Global sets (Fig. 1). As the dataset is highly diverse, with modern varieties (released 1960–2009, *n* = 2294), landraces (1800–1959, *n* = 965), and uncategorized/landrace germplasm (*n* = 1247), we conclude that NDM is representative of NW European wheat germplasm.

### Haplotypic diversity among founders

Haplotypes are sequences of linked genetic variants that are inherited together. In crop genetics, the term haplotype has at least three common usages: (1) long genomic blocks that tend to be found intact despite opportunities to break them apart by recombination. There is some evidence that breeders retain longer blocks than would be expected by chance [23], probably due to selection against recombinants that break apart multiple co-adapted or positive effect alleles at adjacent loci [22]. (2) Genomic blocks of intermediate length that were very recently inherited from the same parents. In the next section, we use this haplotype definition for the NDM RILs, whose genomes are mosaics of founder haplotype blocks, recombined solely by meioses that arose while breeding the lines (we refer to these as “founder haplotypes”). (3) Small blocks of variants that are co-inherited because they are rarely be broken up by recombination over long periods of time. This last definition suggests genomic blocks that are similar because they coalesce at some point in the past and therefore indicates relatedness between samples at different loci [24].

**Fig. 2:**
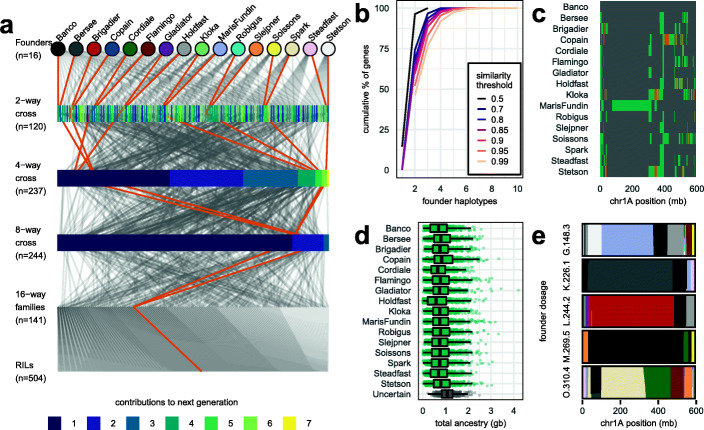
NDM population design and haplotypic diversity. **a** Pedigree showing the construction of 504 recombinant inbred lines (RILs). One exemplar pedigree is highlighted to show how all 16 founders are intercrossed into each RIL. **b** Haplotypic diversity among founders at 73,982 promoter-gene loci with SNP variation, where founders with the same haplotype all have genotypic similarity fractions that exceed the corresponding threshold. **c** Pairwise similarity/dissimilarity between founders on chromosome 1A, determined using a dynamic programming algorithm to infer founder similarity and breakpoint position. Founders inferred to be similar to one another in a given region are the same color. **d** The total length of genomic blocks in NDM RILs inferred to derive from each founder; uncertain ancestry blocks have a maximum founder dosage of < 90%. **e** Inferred founder dosage and ancestry mosaics across chromosome 1A for five representative NDM RILs, with founders coloured as in **a**

We estimated this latter type of haplotypic diversity among the founders using the 1.13 M SNPs called in promoter and genic regions, at two length scales. Both analyses found only limited haplotype diversity. First, to give a gene-centric view, we defined haplotype blocks as the gene regions, plus the promoter associated with each gene. Within each gene-promoter locus, we identified haplotypes shared between founders using complete-link clustering. We defined two founders to carry the same haplotype when their genotypic similarity exceeded 95%. Of 73,982 gene-promoter loci that had SNPs, 38,535 loci (52% of loci with SNPs) had only two haplotypes, 61,438 loci (83%) had at most three haplotypes, and 70,602 loci (95%) had at most four (Fig. 2b).

A limitation of this gene-centric analysis is that it does not extend haplotypes beyond single genes, even when neighboring genes should be part of the same haplotype. Therefore, a second length scale for founder haplotype blocks was determined using a dynamic programming algorithm which automatically set block boundaries so as to maximize similarity between pairs of founders within a block while minimizing the number of block breakpoints, thereby balancing haplotype transitions against calling identical versus non-identical haplotypes. Thus, where the same patterns of haplotype sharing covered adjacent genes, the algorithm extended block boundaries to the maximum degree. Figure 2c shows the pattern of similarity/dissimilarity inferred between pairs of founders on chromosome 1A. Over a wide range of parameters for defining block boundaries, the average number of haplotypes present at any locus rarely exceeded two (Additional file 2: Figure S3; 81.2% of 1.13 M SNP sites inferred to have just two haplotypes). This analysis found slightly fewer haplotypes than the gene-based analysis because it inferred one haplotype (4.1% of sites) when nearby variation was inconsistent, and occasionally split genes with high haplotypic diversity into multiple blocks.

### The NIAB Diverse MAGIC population

We bred a total of 596 recombinant inbred lines (RILs), each descended from all 16 founders via a crossing funnel (Fig. 2a). After 6 generations of inbreeding, all 596 RILs were initially genotyped using the Axiom 35 k wheat breeders’ SNP genotyping array [7]. We called SNPs at 20,688 sites, of which 5747 overlapped with the 1.13 M SNP calls made in the founders. These overlapping sites suggested that only 59.8% of genotyping array probes could have been unambiguously placed using BLASTn [25], underlining the difficulty of using short array probes in polyploids (Additional file 1: Table S2). Therefore, we only used the 5 k overlapping sites as a truth genotype set to find sample misidentifications and estimate the accuracy of sequence-based genotyping in the RILs.

We excluded 46 RILs excessively similar (> 92%) to other RILs, indicating possible errors during population development. We sequenced the remaining 550 RILs after 7 generations of inbreeding by low-coverage WGS (mean 0.304×), and called variants at the 1.13M founder SNP sites from sequence alignments. A further 46 RILs were excluded as their genotypic concordance with the initial 35 k array data was below 95%, leaving 504 RILs in 141 families (RILs in the same “family” are derived from the same 16-way cross), from which we based our main analyses.

We imputed RIL genotypes using STITCH [26] to infer the founder haplotype dosage carried by each line at each location. Figure 2e shows the inferred mosaics of founder haplotypes across chromosome 1A in 5 example RILs. As expected, most recombination is located towards the distal ends of the chromosomes [3]. Founder haplotypes could be confidently assigned (i.e., with > 90% dosage from a single founder) at over 92.2% of sites (Fig. 2d). These founder haplotype assignments implied that an average of 4.8–13.7 recombination events occurred per RIL per chromosome (mean 8.7 sd 2), giving an average of 183 (sd 36.3) recombination events per RIL in total. Consistent with estimated genetic map lengths of 35–37.4M [7, 27], 4.9–5.2 recombination events were observed per Morgan, in line with the predicted ~ 5-fold increase in 16-parent MAGIC populations compared to two-way crosses [28].

The “founder haplotype” blocks found in each RIL are long relative to the length scale for haplotype identity in the founders (Additional file 2: Figure S3). That is, recombination during the 69 years of breeding that separates the founders has meant that haplotypic recombination occurs at a finer genomic scale among the founders than in the MAGIC RILs, whose genomes are an experimentally created recent mosaic of the founder genomes. All 16 founders are distinguishable from one another in the MAGIC RILs. Thus, it was necessary to specify in STITCH that 16 unique founder haplotypes were segregating in order to obtain the highest imputation accuracy (Additional file 2: Figure S3).

**Fig. 3:**
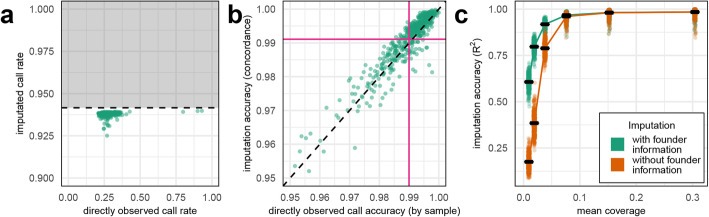
Call rate and accuracy of genotypes after imputation and after downsampling. **a** Imputed call rate (*y*-axis) vs direct call rate (*x*-axis). Only 28.1% of the 1,131,251 SNP sites can be genotyped directly from the low-coverage sequence data, whereas 93.8% of sites had genotypes after imputation. 5.8% of sites (grey region and horizontal dashed line in **a)** were removed by quality control filters after imputation. **b**,**c** Accuracy is defined as concordance at 5747 sites that overlap with the Axiom 35 k array. **c** Imputation before/after downsampling was performed with (green) and without (orange) using the genotypes of the founders as a reference panel

We imputed RIL genotypes with high accuracy and call rates. The fraction of the 1.13 M SNPs that could be called directly from aligned sequencing reads (i.e., without imputation) for 501 RILs varied between 20.9 and 42.7% (mean 27.8% sd 3.4%), as expected for 0.3X-coverage sequence data. A further three RILs were sequenced to higher depth (2.7×, 4.0×, and 4.3×) and had call rates of 79.9%, 90.0%, and 93.0%, respectively (Fig. 3a). After imputation, 94.2% of the 1.13 M SNPs (i.e., 1.07 M) were called across all 504 RILs and the effective call rate of imputed sites was 99.6%. 5.8% of SNP sites were inaccessible or removed by quality control; 0.93% of sites are on the “Un” chromosome in the wheat reference (excluded from imputation), 1.36% were removed by imputation QC (info score < 0.4), and 3.52% had imputed minor allele frequencies below 2.5% and/or missingness above 90%.

Figure 3b shows that the concordance between array and imputed genotypes (AI) and between array and directly called genotypes (AD) are strongly correlated, suggesting that instances of poorer concordance are unlikely to be caused by imputation. Overall, imputation only marginally improved accuracy versus direct calls (mean AI 99.1% versus mean AD 99.0%) but it increased the call rate threefold. Downsampling read coverage showed that genotypes could be accurately inferred from coverage as low as 0.076× per sample (Fig. 3c); above this level, imputation accuracy was independent of whether founder haplotypes were included as a reference panel (mean AI 98.7%) or ignored (mean AI 98.5%).

### Introgressions and segregation bias

Several studies have used genomic data (e.g., SNP density [5]) to map introgressions into hexaploid bread wheat from the secondary and tertiary gene pool [5, 9, 29]. We examined evidence for previously reported introgressions [5, 30–32] using abnormal coverage and non-reference allele frequency in the founders, combined with segregation bias in the RILs, which often accompanies wheat introgressions [15, 33]. We found evidence for at least six introgressions covering ~ 1.1Gb segregating in the NDM, five of which showed segregation bias (Additional file 1: Table S3). For example, the founder Maris Fundin carries a large introgression (640 Mb) from *Triticum timopheevi* on chromosome 2B that inflates the total number of SNPs called on chromosome 2B, relative to the other chromosomes (Additional file 2: Figure S1), and is substantially over-represented among RILs, as expected [33].

### Phenotypic characterization and QTL mapping

We measured 46 phenotypes in replicated field trials over 2 years (Table 1, Additional file 1: Tables S4, S5, and S6), including the 10 time points at which green leaf area (GLA) was measured. In total, 25 phenotypes were collected in both years and two were measured in smaller 1 × 1 m nursery plots (Juvenile Growth Habit, JGH, and Yellow Rust infection, YR, which was also assessed in field trials) to give a total of 73 phenotypic measurements. Phenotype distributions are shown in Additional file 2: Figure S4, showing transgressive segregation for almost all phenotypes. The RIL maximum ≥ founder maximum for 61/73 phenotypes and RIL minimum ≤ founder minimum for 68/73 phenotypes. The minimum yield of the RILs (4.20 and 3.97 t ha^−1^ in year 1 and year 2, respectively) is substantially lower than that of the founders (5.43 and 5.10 t ha^−1^) whereas the maximum yield in the RILs (8.37 and 8.46 t ha^−1^) only slightly exceeds that of the founders (8.11 and 8.28 t ha^−1^). This suggests that recombination in the absence of selection is more likely to break up the favorable allelic combinations that have been selected for in the founders, rather than creating new beneficial combinations. Pleiotropy is common: all phenotypes have significant (*p* < 0.05, Pearson’s correlation test) correlations with at least one other phenotype (Additional file 2: Figure S5).

**Table 1:** Phenotypes collected

Abbreviation	Trait	Abbreviation	Trait
BIS	Basal infertile spikelets	GS39	Flag leaf emergence date
EL	Ear length	GS55	Ear emergence date
ETA	Ear taper	GS65	Anthesis date
ETS	Ear tip sterility	GW	Grain width
EW	Ear weight	GY	Yield
FLA	Flag leaf angle	HEB	Height to ear base
FLED	Flag leaf to ear distance	HET	Height to ear tip
FLF	Flag leaf floppiness	HFLB	Height to flag leaf base
FLL	Flag leaf length	JGH	Juvenile growth habit (Nursery)
FLS	Flag leaf senescence	LOD	Lodging
FLW	Flag leaf width	PHS	Pre-harvest sprouting
GA	Grain area	PIG	General pigmentation
GL	Grain length	SH	Spring habit
GLA#	Green leaf area (10 time points)	SPIG	Stem pigmentation
GLAU	Glaucousity	SW	Specific weight
GPC	Grain protein content	TGW	Thousand grain weight
GPE	Grains per ear	TIS	Tip infertile spikelets
GPS	Grains per spikelet	TS	Total spikelets
GR	Germination rate	YR	Yellow rust infection (Field and Nursery)

From the 1.07 M SNPs imputed in the RILs, we selected a subset of 55,067 pruned by linkage disequilibrium (LD) for QTL mapping. The pruned SNP set tags all other SNPs at *R*^2^ > 0.99. Using genome-wide association scans (GWAS) on both SNP and founder haplotype data, we mapped 136 QTLs across the 73 phenotype/year combinations that were genome-wide significant at the 5% level (a study-wide false discovery rate of 2.6%). Many QTLs for different phenotypes overlapped each other, clustering into 42 distinct genome locations. For 25 phenotypes that were measured in both years, we found 48 QTLs in year 1 and 49 QTLs in year 2, of which 28 were mapped to the same location and were genome-wide significant in both years. For example, in replicated trials lacking fungicide treatment, we mapped yellow rust (*Puccinia striiformis*) susceptibility to four QTLs in year 2 (on chromosomes 2A [30, 34], 2B [35], 3B, and 6A), of which three were also mapped in year 1 (2A, 3B, and 6A); only one (6A) was also mapped in nursery plots treated with fungicide.

In total, 126/136 QTLs at 40/42 genomic locations were mapped using SNP-based associations, whereas only 87/136 QTLs at 30/42 genomic locations were mapped using founder-haplotype-based association tests, which can reveal multi-allelic effects on trait variation, even if the underlying causal variants are not observed directly, albeit at reduced statistical power. Only 10 QTLs at two loci were identified solely by founder-haplotype-based association whereas 49 QTLs and 12 genomic locations were detected by SNP-based association only. The relative scarcity of evidence for multi-allelic QTL effects is consistent with the effectively biallelic gene-level haplotypic diversity observed among the founders.

**Fig. 4:**
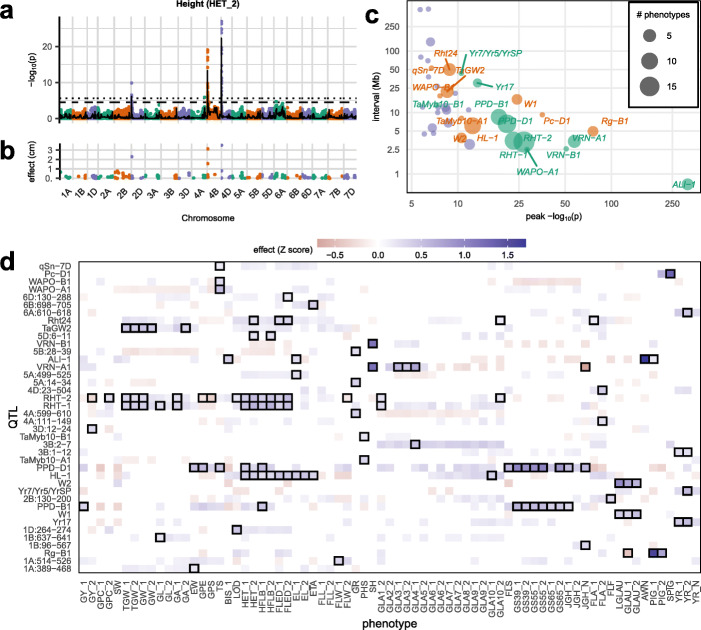
Genotype-phenotype associations. **a** Exemplar Manhattan plot of the genome-wide −log_10_ *p* values of association (logP) between the height to ear tip phenotype from year 2 (HET_2) and 55,067 LD-pruned SNP dosages (dots) or founder haplotype dosages (line). The horizontal lines show the 5% genome-wide significance thresholds for SNPs (dotted) and founder haplotypes (dashed). **b** The 193 non-zero LASSO SNP effect estimates for HET_2. **c** The 40 genomic locations where genome-wide significant SNP associations were found for at least one phenotype, classified by effect size (logP; *x*-axis) and genomic interval width (Mb; *y*-axis). Each circle represents one locus, and its size shows the number overlapping QTLs; the smallest interval width and most significant logP is shown where there are multiple overlapping phenotype associations. Labels indicate QTLs that colocalize with previously described QTLs or candidate genes; green indicates high-confidence colocalization (*n* = 11) and purple low-confidence colocalization (*n* = 10). **d** Pleiotropy across 40 loci: those loci without official names are labelled by chromosome and position in Mb, and 73 phenotypes. Shades indicate the significant (*p* < 0.05) locus phenotypic effects expressed as the number of standard deviations (*Z*-score). Genome-wide significant QTLs are highlighted with boxes

We created a genotype-phenotype map for community use by anchoring all QTLs on the physical map (Additional file 1: Table S7). The median QTL interval length was 9.2 Mb. Figure 4c summarizes the 40 loci with genome-wide significant SNP-based associations. We were able to assign 21 of these, including most of those with the strongest effects, to previously reported QTLs. In 11 high-confidence cases, candidate genes have been reported and/or validated experimentally. In other cases, QTLs either contained homeologs or paralogs of these high-confidence candidates, or previous studies had reported associations to a genetic map using marker data but had not firmly anchored these loci on the reference genome assembly (low-confidence colocalization, *n* = 10). To check mapping interval calibration, we confirmed that six high-confidence candidate loci with annotated reference genome locations (*RHT-1* [36], *RHT-2* [37], *WAPO-A1* [38], *ALI-1* [39]*, TaMyb10-B1* [40], *Yr7*/*Yr5*/*YrSP* [35], *PPD-D1* [41]) were within our mapping intervals.

It is to be expected that most loci with strong effects colocalize with previously reported QTLs, since these large effects are commonly associated with adaptation of the founders to their individual geographic and temporal ranges, and therefore segregate in the NDM. For example, the early flowering allele at the photoperiod locus *PPD-D1* carried by the founder Soissons is favored in southern Europe to avoid the summer drought. The modern semi-dwarfing alleles at *RHT-B1* or *RHT-D1* that have been favored globally since the Green Revolution are absent from founders Banco, Bersee, Copain, Flamingo, Holdfast, Kloka, Spark, Steadfast, and Stetson.

Consistent with the extensive phenotypic correlations (Additional file 2: Figure S5), many QTLs are pleiotropic. The 136 genome-wide significant associations overlap to form only 42 distinct loci. To investigate pleiotropy further, we tested the most strongly associated SNP at each QTL for associations with all other phenotypes, requiring a lower threshold for evidence of association (*p* < 0.05) than was initially used to establish genome-wide significance (Fig. 4d). This suggests that weaker pleiotropy extends beyond the overlap of genome-wide significant associations.

### Gene deletions

We next tested if gene deletions might be causal for QTLs. Our analysis of SNP variation ignored sites that could not be called reliably in all 16 founders, possibly due to whole-gene deletions relative to the reference genome. We observed no sequence coverage in at least one founder at 8019 (7.2%) of genic regions and 1095 (1.1%) of promoter-gene pairs, suggesting possible structural variations (Additional file 2: Figure S1). Based on the deviation in gene coverage from that expected given the mean coverage for the founder, we computed a quantitative gene deletion score (GDS) for each gene and each founder, and imputed these scores into the RILs using the “founder haplotype” ancestry mosaics. We then tested the association between each imputed GDS and each phenotype in order to identify potential causal deletions. Across 27/73 phenotypes, we found 30 GDS associations with *p* values < 10^−6^ (Additional file 1: Table S8). Significant associations always occurred within QTLs previously mapped by SNP association, so this analysis only identified candidate genes with deletion status consistent with the pattern of action across the founders of a QTL. Of these, at 10 loci the peak GDS logP association was at least 90% of the peak SNP logP. The other QTLs we mapped are unlikely to be caused by gene deletions.

Our gene deletion methodology is based on empirical read coverage, and so is likely to be affected by stochastic experimental variations. Hence, it is possible that the association at a true causal deletion might appear weaker than that of a tagging SNP. Another possibility is that multi-allelic structural variants at the same locus, perhaps driven by mobile elements, might weaken the deletion signal, should only one structural variant allele among many be causal for the QTL. Furthermore, deletions are always inferred relative to the reference genome of Chinese Spring, such that insertions or functional genes missing from the reference genome annotation will not be captured in this analysis.

In summary, while gene deletions might be responsible for some of the QTLs, we have not found overwhelmingly strong evidence supporting the deletion of any particular candidate gene, to the exclusion of causal SNP effects. Presence and absence variation may be more reliably inferred when founder genome assemblies become available, as has been used to identify QTLs not found using SNP-based association mapping in *Brassica napus* [42].

### Genomic prediction

We next performed phenotypic prediction using all 55,067 tagging SNPs, to explore the potential for genetic improvement within the NDM. We trained genomic prediction models using three shrinkage methods: ridge regression (RR), least absolute shrinkage and selection operator (LASSO), and elastic nets (EN), using 50 replicates of cross validation. Within each replicate, a randomly selected training set comprising 90% of RILs was used to train a model, which was evaluated on the out-of-sample test set comprising the remaining 10%. We report the average prediction accuracy across 50 replicates. We found that LASSO and EN had almost identical prediction accuracies but EN used on average 26% more SNPs than LASSO (Additional file 2: Figure S6). Accordingly, we only report the LASSO results.

**Fig. 5:**
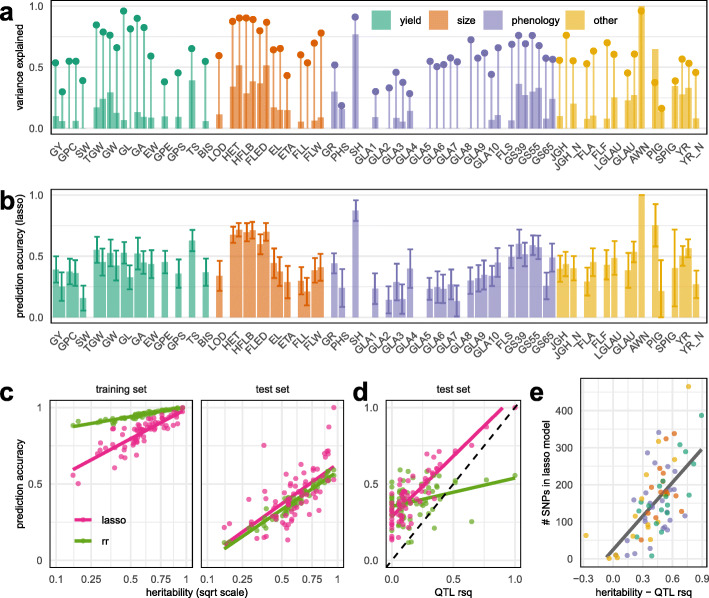
Genetic architectures of 73 trait/year combinations (47 distinct phenotypes) as revealed by QTL mapping and genomic prediction. **a** Phenotypic variation explained by all genome-wide significant QTLs (thick bars) and by the full SNP-based genetic relationship matrix (heritability, thin bars and dots). Phenotypes measured in year 1 and year 2 are paired, shifted to the left and right, respectively. **b** LASSO prediction accuracy (Pearson correlation) across 50-fold cross validation; error bars show sds. **c**, **d** Prediction accuracy correlations (*y*-axis) *vs* sqrt (heritability) or QTL R^2^ (*x*-axis) in the test and training sets under ridge regression (rr) and LASSO genomic prediction models. Prediction into the test set is generally higher with LASSO, especially for traits where more variation is explained by genome-wide significant QTLs (**d**). **e** LASSO models usually include more SNPs when more heritable variation is unaccounted by genome-wide significant QTLs (*x*-axis is difference between heritability and QTL *R*^2^).

LASSO prediction accuracies for all traits are shown in Fig. 5b, alongside the proportion of heritable variation explained by QTLs (Fig. 5a). Across traits, LASSO had higher average out-of-sample prediction accuracy than RR (Fig. 5c), particularly for phenotypes where a larger fraction of variation can be explained by genome-wide significant QTLs (Fig. 5d). LASSO prediction accuracies (correlation coefficients) varied from 0.13 to 1 (mean 0.43) across phenotypes, using models with 1–465 SNPs (mean 155 SNPs). The number of SNPs in the LASSO model is higher for phenotypes where the overall heritability estimate greatly exceeds the fraction of variation that can be explained by genome-wide significant QTLs (Fig. 5e).

Out-of-sample test set prediction confirms that polygenic LASSO SNPs have predictive power and are therefore likely to be tagging genetic variants affecting phenotypic variation. For most phenotypes the LASSO models were polygenic mixtures of a few large effect and many smaller effect loci. A typical example (for height) of the 193 non-zero LASSO SNP effects is shown in Fig. 4c. In contrast, the Mendelian AWN phenotype is fully explained and predicted using a single genome-wide significant QTL.

**Fig. 6:**
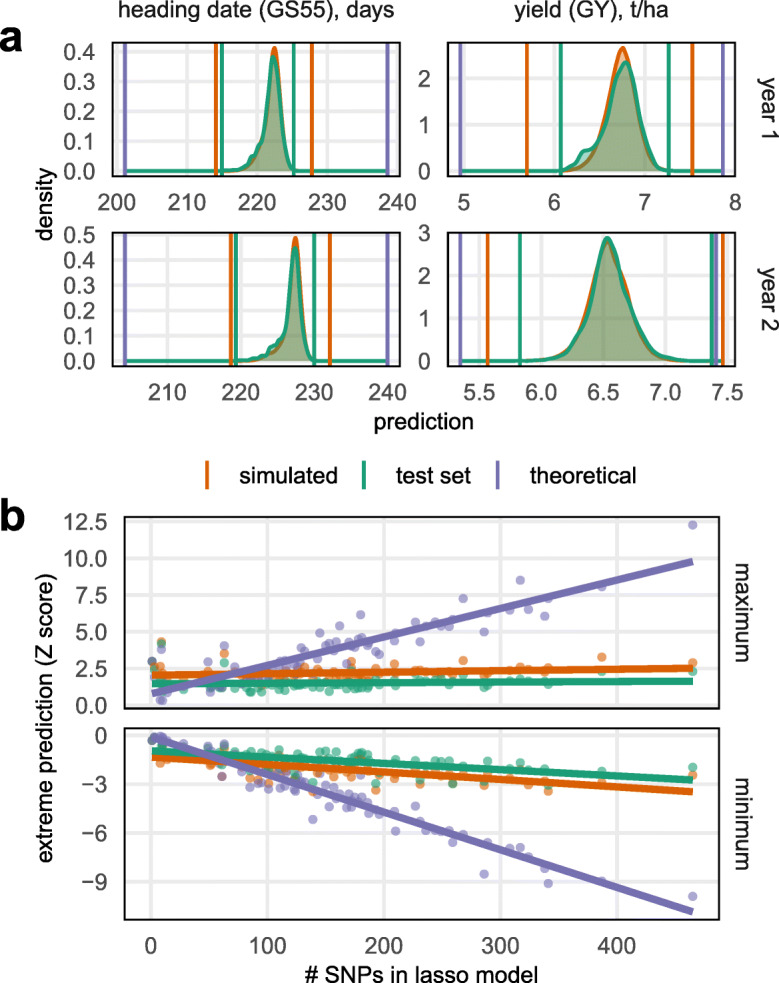
Predicted potential for phenotypic change. **a** LASSO-predicted phenotypes of 504 existing RILs (green distribution), and for 20,160 simulated RILs (orange distribution). Upper graphs: predictions based on year 1 phenotype; lower graphs: predictions based on year 2 phenotype. **b** Estimated theoretical extremes of phenotype predictions possible from the LASSO genomic prediction models (purple, also plotted in **a**), and phenotype predictions in existing (orange) and simulated (orange) RILs, all plotted against the number of SNPs in each phenotype’s LASSO model. Points represent individual phenotypes, lines are linear regressions.

We used the LASSO genomic prediction models to explore the potential for selection in a much larger simulated population of 20,160 MAGIC RILs, 40 times larger than the real population. The simulated RILs were created by permuting the founder identities within the founder haplotype mosaics inferred in the real RILs, preserving linkage through the genetic map. Phenotypes were predicted for the test set of real RILs (10% of all lines) and in the simulated RILs for all 50 prediction models (training/test set resamples). Figure 6a shows, for two representative examples, that the distribution of predicted phenotypes is almost identical in the real (test set) and simulated RILs. Predicted phenotype distributions are less extreme than those measured in the population due to shrinkage in estimating SNP effects.

Next, we predicted the most extreme phenotypic values it would be possible to create in theory from existing segregating variation, should unlimited recombination be possible. That is, we computed the phenotypic prediction in an imaginary line that carries all the alleles predicted to increase/decrease each phenotype. For this exercise, we trained the LASSO prediction models on the full set of 504 RILs so they differ slightly from those used to predict phenotypes in the test set.

Figure 6b shows that the theoretical maximum/minimum phenotypic prediction is linearly related to the complexity of the LASSO model (i.e., the number of non-zero SNP coefficients in the model). Thus, large deviations from the current population mean are predicted to be possible but only through the fixation of a large number of loci, with less potential for change predicted at less-highly polygenic traits. However, none of the 20,160 simulated lines approach these theoretical limits for highly polygenic traits. On average, the lowest/highest phenotypic prediction in the simulated population of 20,160 is only − 0.5 (for the minimum) and + 0.68 (for the maximum) standard deviations lower/higher than the trait predictions in the real dataset of 504 lines. This suggests that hundreds of loci would need to be selected over multiple generations to generate any large phenotypic shifts, in line with the decades of breeding that has been required to produce genetic gain.

### Yield-protein trade-off

**Fig. 7:**
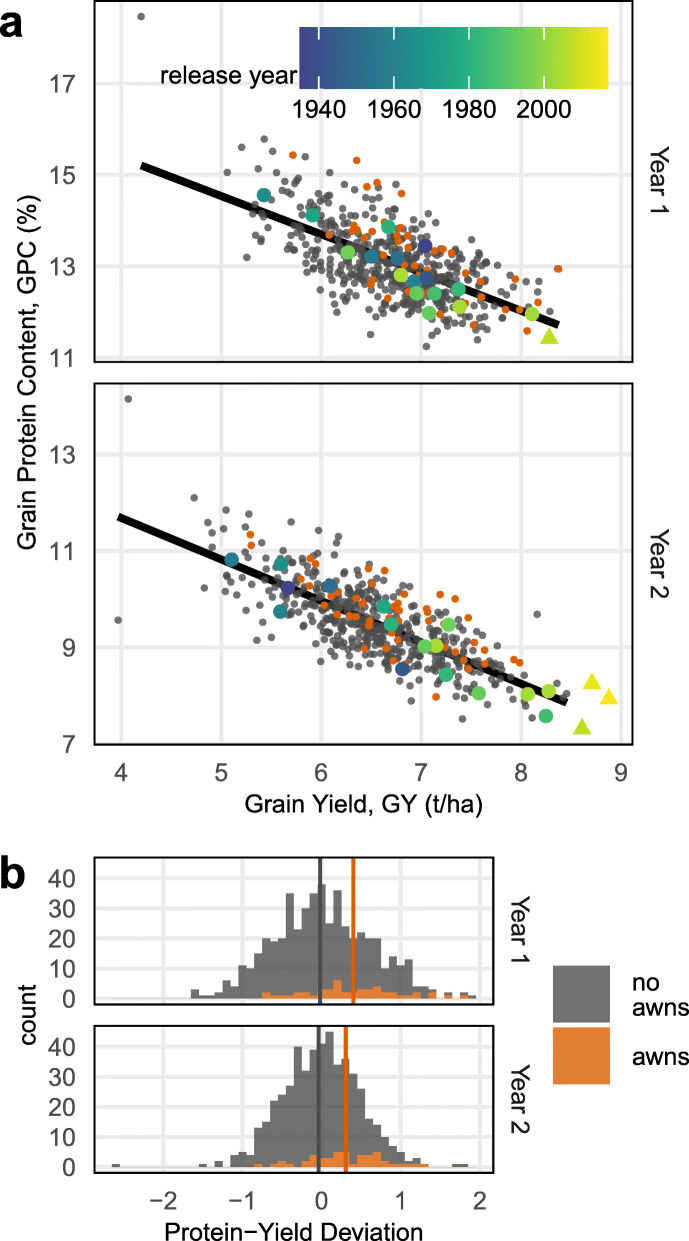
Negative trade-off across 2 years between grain yield (GY) and grain protein content (GPC) in 504 RILs with awns (orange) and without awns (grey). **a** Scatter plots of GY (*x*-axis) vs GPC (*y*-axis), includes 16 founders and 3 recent varieties (triangles, only one measured in year 1) colored by release year. **b** The distribution of protein-yield deviation (PYD): the perpendicular distance from the symmetrical Thiel-Sen regression between GY and GPC, after *Z*-score normalization

For most crops, a trade-off is evident between yield and quality (here defined as percentage protein content), a problem that is well-known in wheat [43, 44]. Thus, identifying opportunities to break this trade-off is important. We estimate that yield has increased by 0.021 t ha^−1^ year^−1^ based on a regression of average yield on founder release year (*p* = 0.006, *n* = 16). However, high average grain yield (GY) is correlated with low average grain protein content (GPC) among the founders (Pearson’s correlation coefficient − 0.94, *p* < 0.001, *n* = 16), Fig. 7a. Although founder genetic material is reshuffled without selection in the RILs, the GY-GPC trade-off is unbroken (correlation − 0.77, *p* < 0.001, *n* = 504), suggesting antagonistic pleiotropy in the underlying genetic effects.

To investigate if any segregating genetic variation might break the trade-off between yield and protein, we defined a new phenotype that combines protein concentration and yield. The protein-yield deviation (PYD) is the perpendicular distance from the symmetrical Thiel-Sen regression between GPC and GY, after *Z*-score normalization. Thus, lines with positive PYD fall above the negative trend between yield and protein. The distribution of PYD is shown in Fig. 7b. The heritability for PYD was 0.41 in year 1 and 0.25 in year 2 and could be predicted with accuracy of 0.26 (sd 0.11) in year 1 and 0.13 (sd 0.11) in year 2. These estimates are lower than those for GY and GPC analyzed separately (GY heritability 0.54 and 0.30, prediction accuracy 0.39 and 0.25; GPC heritability 0.55 and 0.55, prediction accuracy 0.375 and 0.36, Fig. 5). PYD of the founders did not correlate with release date, but these results suggest modest potential to break the yield-protein trade-off in the future, requiring strong and targeted breeding effort, potentially by creating varieties with improved nitrogen use efficiency [45].

We identified one potentially interesting factor increasing PYD, namely the presence of awns. In our population, awns can be predicted with complete accuracy by a tagging SNP at chromosome 5A:698507946 (*n* = 504, *R*^2^ = 1) and other closely linked SNPs. Mixed model genetic association of PYD produced suggestive, but not genome-wide significant, QTLs at this locus in both years (mixed model logP = 3.927, 4.658 in each year). These genetic associations are not conclusive because of the need to adjust for genome-wide significance (PYD thresholds at the 1% level of genome-wide significance are 6.482, 6.373 in each year). However, when interpreted solely as a phenotypic association with awns, the evidence appears stronger (Fig. 7). The linear regression of PYD on awns had logP = 5.13 in year 1 (*n* = 504, *R*^2^ = 0.04) and logP = 5.26 in year 2 (*n* = 502, *R*^2^ = 0.04). Moreover, using Fisher’s method to combine the *p* values from genome-wide association across both years gives a composite logP = 7.269, which exceeds both 1% significance thresholds (Additional file 2: Figure S7). No other locus or phenotype shows such a strong association with PYD.

## Discussion

We have shown that wheat genotypes can be reliably imputed from population WGS with average per-sample coverage as low as 0.075×–0.3×, even without reference panels [26], despite the wheat genome being large, polyploid, and repetitive. We imputed genotypes and founder haplotypes at > 1 M SNP sites in > 500 NDM RILs, which proved ample for genetic mapping and genomic prediction. We therefore recommend imputation from low-coverage WGS as a cost-effective and straightforward genotyping strategy for crops.

Based on SNPs called from genic and promoter capture on the 16 founders, no more than three haplotypes segregate at most genes. Indeed, most genes are biallelic at most: there is no detectable variation at about a quarter of genes on the A and B subgenomes, and at about half on the D subgenome. Nonetheless, the founders were chosen to capture genetic diversity from a panel of UK varieties released since 1935 and are representative of diverse NW European bread wheats. Our results suggest that deeper sampling from this gene pool will reveal few novel haplotypes. For comparison, much greater gene-level haplotype diversity was observed among the 19 founders of the *Arabidopsis thaliana* MAGIC population (4.8 haplotypes per protein sequence, on average), which were global samples from a natural population. Limited haplotypic variation in the UK wheat gene pool probably results from historical selection and population bottlenecks preceding the onset of twentieth century breeding programs [46], as well as the close relatedness among breeding materials in more recent NW European wheat pedigrees [23].

Complete re-assembly and re-annotation of the 16 founders might yield more complete insights into the extent and impact of coding and regulatory variation: we could only make partial functional predictions from genic/promoter capture alone. Greater haplotypic diversity was recently reported among 15 wheat assemblies [4, 22]. These assemblies for diverse global lines suggest that greater haplotypic diversity is available in the wider germplasm. Compared to a SNP-based method, the method of comparison between genome assemblies [4, 22] is probably more sensitive to small sequence differences and therefore measures more recent haplotypic relationships. However, the relationship between haplotypic diversity and phenotypic variation is of most relevance to breeding. It is therefore noteworthy that founder-haplotype-based QTL mapping in the NDM—which has the power to reveal phenotypic differences between founder gene alleles even in the absence of observed variations—largely failed to improve over biallelic SNP-based mapping. We conclude that the NDM is for the most part functionally biallelic and therefore differs from other multi-parent populations. For example, 40% of QTLs identified in a heterogeneous stock of rats were attributed to multi-allelic/haplotypic effects [47].

Across phenotypes, we found a wide range of underlying genetic architectures. For a few simple phenotypes, large fractions of the phenotypic variance could be explained through one or two QTLs, but most quantitative traits, including yield, were polygenic with QTLs of smaller effect (Fig. 5a). Most QTLs tended to have pleiotropic effects and therefore naïve selection on one phenotype is likely to induce correlated responses in other phenotypes. Notably, directed selection considering these effects might go some way towards breaking the yield-protein trade-off [43, 44], on which there appears to have been little progress among the founder varieties. Using evidence from both study years, we found that the awn phenotype and its causal locus [39] was associated with deviations above the negative trend line between yield and protein. Awns have a plausible photosynthetic role during grain filling [48] and may increase protein content [49]. Thus, if the association is replicated in elite germplasm, the awn locus and phenotype might be targeted towards simultaneously increasing yield and protein content. Such relationships between phenotypes are more easily interpreted in populations with controlled structure, like NDM [13]. Unlike previous wheat MAGIC studies that focus on a smaller number of phenotypes, including awns [15], the multi-phenotype approach taken here has uncovered novel phenotype-phenotype associations and allows direct comparison between the genetic architectures of different phenotypes.

We compared the accuracy of ridge regression, LASSO, and elastic net genomic prediction models across phenotypes. The prediction accuracy of ridge regression is correlated with trait heritability (Fig. 5c), as expected given that ridge regression is equivalent the mixed model GBLUP used to estimate heritability [50, 51]. The accuracy of LASSO and elastic net were very similar, but LASSO was more parsimonious in using fewer SNPs in the prediction model (Additional file 2: Figure S6). These feature selection methods tend to apply less shrinkage to individual SNP effects and therefore perform particularly well for traits where there are QTL with large effects (Fig. 5d). In general, we found LASSO and elastic net performed better than ridge regression for out-of-sample genomic prediction. This may be expected from the population design. The variation in kinship among MAGIC RILs is lower compared to samples from the wider germplasm. Much of the predictive ability of ridge regression results from exploiting kinship rather than from tagging causal variants [52] so there is less opportunity for high prediction accuracy in unstructured populations.

We found that LASSO genomic prediction models could achieve reasonable prediction accuracy with modest numbers of SNPs; the mean out-of-sample prediction accuracy was 0.43, using on average only 155 SNPs per phenotype. Here, rather than using low marker densities, we trained models that select a just a few hundred markers from ~ 55 k tagging SNPs. In part, this may be a consequence of the design and construction of MAGIC populations, which eliminate rare alleles and create blocks of markers inherited from each founder. Previous crop and livestock studies have found very sparse markers can be sufficient for useful genomic prediction [6, 53, 54]. The wider wheat germplasm also tends to have long haplotypic blocks [22] that can be easily tagged in prediction models [10]. These factors may be responsible for the use of far fewer markers than used to generate polygenic prediction scores in humans [55], where there is a long tail of rare variation and less linkage disequilibrium.

We used our LASSO models to predict the potential for future phenotypic change from segregating variation. By simulating a larger NDM population of over 20,000 lines, we showed that blind-breeding a very large population in the hope of generating novel combinations of beneficial alleles is inefficient. As expected, the most extreme predicted values (both maximum and minimum) in the simulated RILs exceed than those in the real dataset because novel allelic combinations are generated in the larger simulated population. However, the average improvement in extrema between the real set and simulated phenotype predictions is only ~ 0.5 standard deviations (Fig. 6c). This is in line with extreme-value theory when applied to the Gaussian distribution. While our models predict larger phenotypic shifts are possible, these require selection on dozens of loci. These results are consistent with the trend towards using genomic selection in crops [56] and confirm the difficulty of breeding to improve polygenic traits. Nonetheless, breeding has consistently improved yields [2].

## Conclusions

The dramatic genetic improvement witnessed over 70 years of wheat breeding has largely been achieved through the fine shuffling of a few haplotypes to recombine polygenic alleles of small effect, combined with the introduction of alien introgressions from wide crosses. There are a small number of loci with large effects on particular phenotypes, notably including the “Green Revolution” semi-dwarfing alleles introduced from Japan [57]. The introgression of large genomic segments from other species has most commonly been for sources of resistance to specific diseases [5, 32, 33]. We predict that future phenotypic change from segregating variation will require selection at many loci. Nevertheless, we predict that segregating genetic variation is available for improving both yield and quality while avoiding negative pleiotropic effects via targeted selection. Breeders now have a choice whether to continue selecting from within the existing variation pool while introgressing selected exotic alleles, or to ambitiously expand the available pool of haplotypic diversity genome-wide.

## Methods

### Population creation

The 16 NDM founders were chosen to capture the greatest genetic diversity using PowerMarker genetic analysis software [58]. They were chosen from 94 NW European wheats released in the UK that were genotyped with 546 DArT and 61 SSR markers; the full panel also included 96 US and 50 Australian varieties, which were excluded based on STRUCTURE analysis [59]. The founder selection process was run iteratively with the varieties “Robigus” and “Soissons” first fixed to be included to coincide with the founders of the 8-founder NIAB Elite MAGIC population [14]. Then the most frequently selected additional 4, then 9, and 12 varieties were fixed in multiple iterative selection runs and finally the most frequently selected 16 were chosen. Seed for the founding varieties was sourced from the John Innes Centre Germplasm Resource Unit (http://www.jic.ac.uk/germplasm/).

These founders were intercrossed in a balanced funnel crossing scheme, based on a Latin square field trial design, over four generations to create 16-way crosses with all the founders equally represented in their pedigree. First, all 120 possible 2-way crosses between founders were made in a half diallel scheme. Two-way plants were then crossed in 60 4-way combinations. Multiple plants from each family were used in crossing from 2-way onwards, in order to maintain maximum founder allelic diversity within the population. 30 crossing combinations were made between 4-way plants to create 8-way crosses, making between five and eight replicate crosses per combination using different plants. These were intercrossed in 15 combinations to create balanced 16-way crosses, with each combination replicated between six and fifteen times using different 8-way plants. This resulted in 174 16-way plants from which one to sixteen inbred lines per 16-way family were made through single seed descent (SSD). In total, 596 RILs were advanced to the F_7_ stage when seed for phenotyping was multiplied in 1 × 1 m nursery plots. Additional file 1: Table S9 gives details the number of plants involved in each cross and Fig. 2a shows the pedigree for the 504 RILs used in our main analysis only.

### Phenotyping

RILs from the population were phenotyped in field trials over multiple environments near Cambridge, UK. Yield trials were conducted in the growing seasons 2016–2017 and 2017–2018, hereafter year 1 and year 2 (phenotype suffix codes _1 and _2). Information on location, soil type, key dates, and inputs for both years are given in Additional file 1: Table S4. Yield plot dimensions were 2 m wide and 4 m long, and plots were sown at a density aiming to achieve 300 plants m^−2^. In year 1, 596 lines were included in two replicates, the sixteen founders in four replicates and the commercial control variety “KWS Santiago” in 24 replicates in a randomized nested block design with 16 main blocks of 80 adjacent plots which comprised each row in the trial and eight sub-blocks of ten plots nested within each main block. In year 2 trials, 596 lines and the 16 founders were included in two and four replicates respectively but three control varieties (“KWS Santiago,” “Skyfall,” and “Shabras”) were all included in four replicates. Plots were again randomized in a nested block design but including additional plots making a larger trial, consisting of 20 main blocks of 115 adjacent plots, which comprised each row, and 23 sub-blocks of five plots nested within each main block.

Disease observation trials (DOTs) were conducted near Cambridge, UK, in the same years as the yield trials to assess resistance to crop diseases. These plots consisted of two 1.2-m length rows, treated with no fungicide but otherwise standard inputs. Due to local conditions, DOTs had a high natural likelihood of yellow rust infection (*Puccinia striiformis* f.sp. *tritici*) and were not experimentally challenged with pathogens. In both years, DOTs included two replicates of 596 RILs, four replicates of the 16 founders, and 68 additional replicates of the susceptible founder “Robigus.” Trial designs included two main blocks of 660 plots, with 11 sub-blocks of 60 plots nested within main blocks. All trial designs for both yield and disease observation trials were made using the package “blocksdesign” in R. Phenotyping of some traits was also carried out in 1 × 1 m seed nursery plots where lines were not replicated but the founders were in three replicates and randomized across the nurseries (phenotype code _N).

A wide range of traits were phenotyped across the field trials, including traits for crop developmental morphology, phenology, plant stature and canopy architecture, yield and yield components such as spike and grain morphology, disease resistance, pigmentation, plant glaucosity, indications of stress response, lodging, grain protein content, and vernalization requirement. A summary of these traits and abbreviations are presented in

Table 1 and details of phenotyping methods are listed in Additional file 1: Table S5.

### Trials analysis

Adjusted phenotype values were calculated as best linear unbiased estimates (BLUEs) for each trait separately for each trial year using mixed effects models with ASRemL [60]. Genotype was considered a fixed effect while experimental blocking structure as well as other covariates such as harvesting day, where relevant, was included as random effects. Spatial models including first- and second-order auto-regressive spatial models were also used. Model simplification was carried out where models with all possible combinations of random effect terms and spatial terms for row and column were run and the best fitting model was chosen based on Akaike Information Criteria (AIC). Model residuals were visually checked for normality and equal variance. The best linear unbiased estimates (BLUEs) for all phenotypes for the 16 founders and for the 504 RILs used in our main analysis (see below) are provided in Additional file 1: Table S6. We used symmetrical Thiel-Sen regression (implemented in the “deming” R package) after phenotype normalization to characterize the relationship between protein content (GPC) and yield (GY). The protein-yield deviation (PYD) phenotype is calculated as the Euclidian distance from this regression line.

### Genotyping array data

All DNA extraction was performed using the Qiagen DNeasy Plant Kit on leaf tissue samples taken from emerging leaves of seedlings. First, genotyping was performed at the Bristol Genomics Facility using the Axiom 35 k wheat breeders’ array [7]. Initially, two 384-sample plates were genotyped. Seed from the plants used as founders were genotyped on each plate (32 samples) along with extra seed from the original varietal seed stock used (28 samples) and seed from founders propagated to 2017 (16 samples). In addition, 596 RILs were genotyped after 5 generations of selfing (F_6_). To account for genotyping failures and to ensure the accuracy of sample labels, 150 RILs were re-genotyped in the F_7_ generation along with a further replicate of each founder.

Genotype calling was performed using the Affymetrix Power Tools (v1.19) and SNPolisher R packages, following the recommended Axiom analysis pipeline. All samples except two-way crosses were given the standard inbreeding penalty, 4, which penalizes calling heterozygous genotypes. Four samples failed the “dish quality control” threshold (0.82) and a further 28 samples with call rates below 97% were excluded. Marker classifications were performed using “ps-classification” and “ps-classification-Supplementary” functions with options --species-type polyploid --hom-ro false. All calls were adjusted using the standard 0.025 confidence threshold using the Ps_CallAdjust function.

Samples were compared to one another using the 14,935 markers classified as “PolyHighResolution” only. Overall, 46 RIL pairs were found to be > 92% similar (mean 98.5% genotype similarity), where all other comparisons between MAGIC lines were, at most, 84% similar (mean 67.8%). These apparently duplicated genotypes could indicate genotyping, labelling, or propagation errors so only one RIL from each pair was used for sequencing (550 RILs). To ensure pedigree accuracy, we chose the RIL in each pair that was genotypically most similar to other RILs derived from the same 16-way cross (i.e., in the same family).

### Sequencing data

For whole-genome sequencing, DNA was extracted from 550 RILs at the F_7_ generation. DNA for RILs that failed quality control were extracted again at the F_8_ generation (*n* = 50). Sequencing and library preparation was performed at Novogene, where libraries were generated from 1.0 μg DNA per sample using the NEBNext DNA Library Prep Kit. Sequencing was performed on a NovaSeq 6000 instrument (Illumina) to get at least 6 Gb of raw sequence data (2 × 150 bp paired end reads) per sample. One founder (Holdfast) was sequenced to 15.8× coverage using the same method.

The other founders were sequenced after capture using two recently designed probe sets targeting promoter and genic regions, respectively [19]. Capture was performed at the Earlham Institute following the SeqCap EZ Library SR v5.1 protocol (Roche NimbleGen Inc., Madison, WI, USA) with 1 μg of genomic DNA sheared to 300 bp [19]. Four captures were performed using 8 samples per set (2× promoter captures and 2× genic captures). Samples for the founder Stetson were included on all four capture experiments, so roughly double the sequence data was obtained for this founder (Additional file 1: Table S1). Sequencing with 2 × 150 bp reads was performed at the Earlham Institute on a NovaSeq 6000 instrument (Illumina) with 16 promoter capture libraries on one lane and 16 genic capture libraries on another lane.

### Variant calls and imputation

All reads were aligned to the bread wheat reference genome from cv. Chinese Spring (RefSeq v1.0) [3] using bwa-mem (version 0.7.12) [61] and sorted using samtools (version 1.3.1) [62], which was also used to calculate coverage. For compatibility with the bam file format, we split each chromosome in the reference genome at the halfway point before alignment. We called variants from the founder sequences within the high-confidence gene, promoter and 5′ UTR regions targeted by the capture probes [19] using GATK (version 4.0.8.0) [63] HaplotypeCaller and GenotypeGVCFs (options --interval-padding 100 --minimum-mapping-quality 30). We used vcftools (version 0.1.15) to include only biallelic single-nucleotide polymorphisms (SNPs) with average coverage depth between 5 and 60 (all per-sample coverages between 2 and 120) and no missing calls. We also filtered with bcftools (version 1.2) [64] using standard quality control options --exclude “QD <2 || FS >60.0 || MQRankSum<-12.5 || ReadPosRankSum<-8.0 || SOR >3.0 || MQ <40”. This left 1.78 M SNPs, of which we only use the 1.13 M sites with no heterozygous calls (--genotype ^het option) for our main analyses.

We first called genotypes in the RILs at these 1.13 M SNP sites directly using GATK HaplotypeCaller in GENOTYPE-GIVEN-ALLELES mode, using the same options as above. We assessed the concordance between array genotypes and these direct calls (AD) at overlapping sites (see below). For 10 RILs, the directly called sequencing variants most closely matched genotyping array data for a different line than expected. These were excluded because the source of the discrepancy (sequence data or array data) cannot be established. The concordance between our genotyping array data and direct calls (AD) was below 95% for a further 36 RILs, which were excluded (mean AD 84.7% for removed lines), leaving 504 RILs. We estimated heterozygosity in these 504 RILs using only genotypes called from at least four reads. Of 2.6 M such genotype calls, only 0.67% were called as heterozygotes.

We imputed genotypes at the 1.13 M SNP sites using the alignments and STITCH software (version 1.5.7) [26]. Because alignments were to a reference genome with chromosomes split in half, we first ran STITCH with the generateInputOnly option, and then joined the input files for each chromosome half before imputation. For all runs, we used the parameters nGen = 3, minRate = 0.001, bqFilter = 30, method = “diploid-inbred” and then filtered all sites with an info score below 0.4, minor allele frequency below 2.5%, or missingness above 10%. For our main analysis, we used the genotype calls in the founders as a reference panel and outputted the estimated ancestry dosages of each founder at each position in each RIL using the outputHaplotypeProbabilities and output_haplotype_dosages options. When using the founders as a reference panel, we removed options that estimate and update the haplotypes in the population (shuffleHaplotypeIterations, reference_shuffleHaplotypeIterations, refillIterations). To test accuracy when reference panels are not available, we re-ran imputation without providing the founder genotypes, using 40 iterations to estimate the haplotype space and recombination mosaics. We also used the downsampleFraction option to randomly sample a fraction of alignments with/without using the founder reference panel. Finally, we tested imputation accuracy (without a reference panel), when fewer than sixteen haplotypes were assumed to segregate in the population by varying the K parameter (Additional file 2: Figure S3).

### Genotype comparisons

For comparison against the sequencing dataset, we used all genotyping array markers. Replicates of founders and MAGIC RILs (where available) were used to make a consensus call where the most common genotype across replicates was taken as the consensus and only retained when more than 50% of the non-missing calls were in agreement. In addition, markers where one homozygous genotype was missing from all RILs were converted such that all heterozygous calls were assumed to be in the missing homozygous class. The failure to detect a homozygous class is likely to be a result of polyploidy, which can reduce differentiation between the three genotype classes and make them hard to distinguish. Finally, to get genome coordinates for the genotyped markers, BLASTn v2.2.30 [25] was used to compare the 75-bp probe sequences (cerealsdb.uk.net) [7] against the reference genome [3]. When matching the SNP array data with the sequenced SNPs, array sites were excluded if there had missing or heterozygous founder calls or if the genotypes and targeted SNP alleles did not match the founder sequence data. We found 5877 sites that overlapped between the genotyping array data and the sequencing data (Additional file 1: Table S2).

To compare against global wheat diversity, we called founder genotypes at 113,457 genotyping array sites that were polymorphic among 4506 diverse global wheat accessions [8]. We called genotypes from alignments with mapping quality scores of at least 30 using GATK HaplotypeCaller in EMIT_ALL_SITES mode with the –emit-ref-confidence BP_RESOLUTION option, providing a bed file of the 113,139 genotyping array sites [8]. We only considered sites where genotypes could be called in all 16 founders (*n* = 56,063). We used genotyping array calls for cv. Chinese Spring to determine reference/non-reference alleles on the genotyping array, ignoring sites called as heterozygous (*n* = 109) or missing (*n* = 306) in Chinese Spring. Seven of the MAGIC founders were also present in the global genotype set (Brigadier, Copain, Maris Fundin, Soissons, Spark, Steadfast, Stetson)^7^. The average concordance of the global genotype calls and our sequencing calls for these founders was 94.3% (sd 0.63%). We excluded 5491 (9.8%) sites that had mismatches across these founders, many of which are likely to reflect differences in the underlying genetic variation picked up by the different genotyping technologies. Two other founder variety names were in the genotyping array dataset^7^ (Banco and Holdfast) but the genotyping calls did not match (concordances 74.2% and 71.4%, respectively), which may reflect differences in the seed stock used.

### Haplotype diversity among founders

First, we used the SNPs called within each promoter-gene pair to estimate haplotypic diversity among the founders. We calculated absolute (Manhattan) pairwise genetic distances between founders at each site and then used complete linkage clustering to define haplotypic groups using dist and hclust functions implemented in R statistical software (version 3.6.0) [65]. This was repeated using different similarity thresholds to define haplotypes.

Second, we determined haplotype breakpoints using a dynamic programming algorithm. For each pairwise founder combination, our algorithm calculates a mosaic of genotypic similarity/dissimilarity akin to the Viterbi path from a hidden Markov model. Genotype matches and mismatches are allocated a score (1 by default). To prevent excessive switching between states, there is also a “transition penalty” for inferring a change between matching and mismatching states. Based on their pairwise matching/mismatching states, we then infer the total number of haplotypes inferred at each site. We repeat this procedure with different transition penalty parameter choices (Additional file 2: Figure S3). Figure 2c shows founder similarity using a transition penalty of 200.

### Genetic mapping and heritability

For mapping, we used the full set of 1,065,185 high-quality SNP sites called in 504 RILs after imputation and quality control filters. From these, we selected a subset of *p* = 55,067 SNPs such that every other SNP was tagged at *R*^2^ > 0.99 by a member of the subset using PLINK (version 1.90) with option --indep-pairwise 500 10 0.99. The genotype dosages at each tagging SNPs were standardized to produce a 504 × 55,067 genotype dosage matrix ***G*** which was used to calculate the genetic relationship matrix (GRM) ***K = GG*** ’ /*p*. The phenotypic variance-covariance matrix for a given vector *y* of standardized phenotype values was modelled as $\boldsymbol{V}=\boldsymbol{K}{\sigma}_g^2+\boldsymbol{I}{\sigma}_e^2$where ${\sigma}_g^2,{\sigma}_e^2$ are the additive genetic and environmental variance components, estimated by maximum-likelihood [66]. The heritability of a trait was defined as ${h}^2={\sigma}_g^2/\left({\sigma}_g^2+{\sigma}_e^2\right)$. The matrix square root of the variance matrix was calculated by eigendecomposition of ***V*** as ***A***^**2**^ ***= V***, and the mixed model transformation of the data performed, i.e., ***y → A***^***−*1**^***y***, ***G → A***^***−*1**^***G***, ***V → I*** , to remove the inflationary effects of unequal relatedness on genetic associations before association mapping.

We performed association tests at the level of both SNPs and founder haplotypes using R statistical software (version 3.6.0 )[65], using purpose-written R scripts available on GitHub (see “Availability of data and materials”). Initially, we tested the null hypothesis of no association at each SNP site in the 55 k tagging SNPs. We then determined genome-wide thresholds for statistical significance using 1000 permutations on the transformed phenotypes, as described in reference [67]. If any association exceeded the 0.05 threshold (smaller *p* value than found across at least 950 phenotypic permutations), then we repeated the association test at all of the ~ 1.1 M SNPs on the chromosome with the strongest association signal (lowest *p* value). Mapping intervals were defined to include SNPs surrounding the peak SNP, with log_10_(*p*) values within *d* units of *x* using *d* = max {2, 0.1*x*} where *x* is the peak log_10_(*p*) value. The interval for founder-haplotype-based tests includes the range of sites that have log_10_(*p*) values within *d* units of *x*. SNP-based intervals were calculated using the same measure but then extended by the minimum of 5 Mb or the distance to the next SNP in either direction that the same “strain distribution pattern” [47] as any highly associated SNPs (SNPs with log_10_(*p*) values within *d* units of *x*). The “strain distribution pattern” is the pattern of major/minor alleles across founders. This procedure is designed to capture the uncertainty in the positioning of relevant recombination events on either side of the QTL peak. We fitted QTLs in a stepwise manner by fitting the phenotype against the most strongly associated SNP (or founder haplotype dosage) whenever genome-wide significant QTLs were detected. The above association test procedure was then repeated using the phenotype residuals after fitting all previously identified QTLs. This allows closely linked QTLs to be detected when they have different patterns of causal variants among RILs. Where QTL associations were found for different genotypes, they were judged to be at the same locus if they had overlapping mapping intervals and at least one matching strain distribution pattern at highly associated SNP sites.

For 40 QTLs identified using SNP-based associations, we looked in the set of ~ 55 k SNPs that were called in 4500 global wheat accessions [8] for markers within the mapping interval that had founder genotype calls consistent with the QTL. Where more than one phenotype measurement was mapped to the same locus, we used the smallest QTL interval for matching. Twenty two QTLs could be “matched” in this way and we can therefore estimate the frequency of these functional variants in the global germplasm (Additional file 2: Figure S2). Where more than one candidate SNP from the set of 55 k could be plausibly matched with the QTL, we used the average global MAF. We evaluated all QTLs to identify potentially causal variants that we estimated to be rare in the global germplasm. The QTLs that are rarest in the global population are for yellow rust resistance (Yr17 on chromosome 2A [30], estimated global MAF 6.7%) and grain yield in year 2 (3D:12–24, estimated global MAF 10.4%). A caveat to this analysis is that the linkage disequilibrium between SNPs and the underlying causal variation could break down in the wider population. Furthermore, the design of genotyping arrays biases them towards the detection of common variation [68]. We are therefore likely to underestimate the degree to which rare functional alleles have been detected in our population.

### Genomic prediction

We evaluated the accuracy of trait prediction within NDM and estimate the extent of polygenic variation beyond genome-wide significant QTLs. We conducted genomic prediction across all phenotypes using three shrinkage-based methods: ridge regression (RR), elastic nets (EN), and least absolute shrinkage and selection operator (LASSO), using the R package glmnet [69], which estimates optimal shrinkage parameters for each genomic prediction method based on the training set. For each method, we conducted 50 rounds of cross validation by randomly sampling 90% of the RILs (*n* = 454) as a training set in each round to train the model, which was then used to predict the remaining 10% of RILs (*n* = 50)—the test set. For the three methods, the model equation can be written generally as *y* = *μ* + ***G****β* + *ε*, where *y* is the estimated trait value, *μ* is the model intercept, *β* is the vector of SNP effects, *G* is the genotype dosage matrix, and *ε* is the residual error. With appropriate choice of ridge parameter $\lambda ={\sigma}_e^2/{\sigma}_g^2$, RR is equivalent to a mixed model in the sense that the RR estimated SNP effects are identical to the mixed model best linear unbiased predictors (BLUPs) [50, 70]. This explains the near perfect correspondence between estimates of heritability and RR prediction accuracy (Fig. 5c).

We then predicted phenotypes in the test set by multiplying all SNP coefficient estimates by their corresponding genotypes in the test set (and adding the intercept term). We reported the training and test set prediction accuracy as the mean Pearson correlation coefficient of the predicted trait values and the actual phenotype values over 50 rounds of cross validation.

We used these genomic prediction models to evaluate the potential for phenotypic change in a simulated NDM population of 20,160 RILs, assuming the same patterns of recombination as actually observed. We did this by simulating new breeding funnels. Thus, we permuted the population founder haplotype identities 40 times across the 504 RILs and then projected the permuted founder genotypes onto the new lines. This creates new genetic combinations while retaining the mosaic breakpoints, genetic map, and linkage disequilibrium found in the real population. We applied the LASSO models (trained as above on the 504 RILs) to predict phenotypes for the simulated MAGIC RILs. We further calculated the theoretical maximum and minimum phenotype values that are possible given the genomic prediction models and the variants segregating in the population, by summing the estimated effects for all positive or negative SNP coefficients, respectively.

### Gene deletion analysis

We asked if gene-level coverage variation among founders might explain phenotypic variation. In each founder *f* and at each gene feature *g*, we computed a gene deletion index *D*_*gf*_ based on the number of reads aligning to the associated capture sequences, normalized by the overall coverage for that founder. The gene deletion score (GDS) for each MAGIC RIL *i* and feature *j* was computed as ${S}_{ij}=\sum \limits_f{H}_{ij f}{D}_{jf}$, where *H*_*ijf*_ is the founder haplotype dosage for founder *f* in RIL *i* at gene *j*, as computed by STITCH. For each phenotype, a mixed model GWAS was performed, using the GDS in place of SNP dosages and with a genetic relationship matrix computed from the GDS (Additional file 1: Table S8). We also repeated the genomic prediction analysis described above by replacing the SNP genotype dosage matrix with the GDS matrix (Additional file 2: Figure S5).

### Introgressions

Evidence for introgressions was based on summary statistics (coverage, non-reference allele frequency in founders and RILs) calculated in 10-Mb windows moved in 5-Mb steps. Within introgressions, carriers should have a high proportion of non-reference alleles due to the alignment of inter-specific genetic material to the bread wheat reference genome. Introgression boundaries were defined by the extent of 10-Mb windows where all introgression carriers had a higher proportion of non-reference alleles than all non-carriers. Within these regions, we then checked the relative coverage of carriers and the extent to which the alleles of carriers are over- or under-represented among the RILs. This evidence is summarized in Additional file 1: Table S3.

## Supplementary Information

**Additional file 1: Table S1.** Summary of NDM founder varieties and sequencing coverage. **Table S2.** Summary of overlap in sites called from wheat breeders’ genotyping array and SNP sites called in founders from sequencing data. **Table S3.** Summary of evidence for introgressions segregating among NDM founders. **Table S4.** Summary of trial inputs and conditions in 2016-17 (year 1) and 2017-18 (year 2). **Table S5.** Description of all phenotype measurement methods and timings. **Table S6.** Best Linear Unbiased Estimate for all phenotypes in 504 RILs. **Table S7.** Summary of all genome-wide significant QTL associations found for SNP-based and Haplotype-based mapping. **Table S8.** Gene-Deletion-Score (GDS) loci with GDS-phenotype associations with logP> 6. **Table S9.** Summary of crossing scheme.

**Additional file 2: Figure S1.** Distribution of SNPs across chromosomes and founders. **Figure S2.** Minor Allele Frequency among ~ 4500 global wheats and the NDM founders. **Figure S3.** Haplotype block length in founders and MAGIC lines. **Figure S4.** Distribution of Best Linear Unbiased Estimates (BLUEs) across 73 phenotypic measurements. **Figure S5.** Correlations between phenotypes in the 504 RILs. **Figure S6.** Comparison between genomic prediction using LASSO and ELNET and between genomic prediction based on SNPs and gene deletion scores. **Figure S7.** Association mapping of the Protein Yield Deviation (PYD) compound phenotype.

**Additional file 3.** Review history.

## Data Availability

The DNA sequence data for this study have been deposited in the European Nucleotide Archive (ENA) at EMBL-EBI under accession number PRJEB39021 (https://www.ebi.ac.uk/ena/browser/view/PRJEB39021) [71]. SNP genotypes are available from the ENA for the founders (accession ERZ1643321, https://www.ebi.ac.uk/ena/browser/view/ERZ1643321) [72] and for the RILs before imputation (accession ERZ1643320, https://www.ebi.ac.uk/ena/browser/view/ERZ1643320) [73] and after imputation (ENA accession ERZ1643322, https://www.ebi.ac.uk/ena/browser/view/ERZ1643322) [74]. The phenotypes and genotyping array genotypes for founders and MAGIC RILs are available in text format from UCL Research Data Repository entry 14388461 https://rdr.ucl.ac.uk/account/articles/14388461, doi:10.5522/04/14388461 [75], and from http://mtweb.cs.ucl.ac.uk/mus/www/MAGICdiverse, which describes the data in more detail. Custom analysis scripts and pipelines are available from https://github.com/michaelfscott/DIVERSE_MAGIC_WHEAT [76]. The remaining datasets supporting the conclusions of this article are included within the article and its additional files. NDM germplasm resources are described at https://www.niab.com/research/agricultural-crop-research/resources.
