# Supplementary material for: Limited haplotype diversity underlies polygenic trait architecture across 70 years of wheat breeding
Source: Genome Biol. 2021 May 6;22:137. doi: 10.1186/s13059-021-02354-7 (PMC8101041; doi:10.1186/s13059-021-02354-7)
Supplement: Supplementary file 2 — Additional file 2: Figure S1. Distribution of SNPs across chromosomes and founders. Figure S2. Minor Allele Frequency among ~ 4500 global wheats and the NDM founders. Figure S3. Haplotype block length in founders and MAGIC lines. Figure S4. Distribution of Best Linear Unbiased Estimates (BLUEs) across 73 phenotypic measurements. Figure S5. Correlations between phenotypes in the 504 RILs. Figure S6. Comparison between genomic prediction using LASSO and ELNET and between genomic prediction based on SNPs and gene deletion scores. Figure S7. Association mapping of the Protein Yield Deviation (PYD) compound phenotype. [file 13059_2021_2354_MOESM2_ESM.pdf]

Supplementary Figures

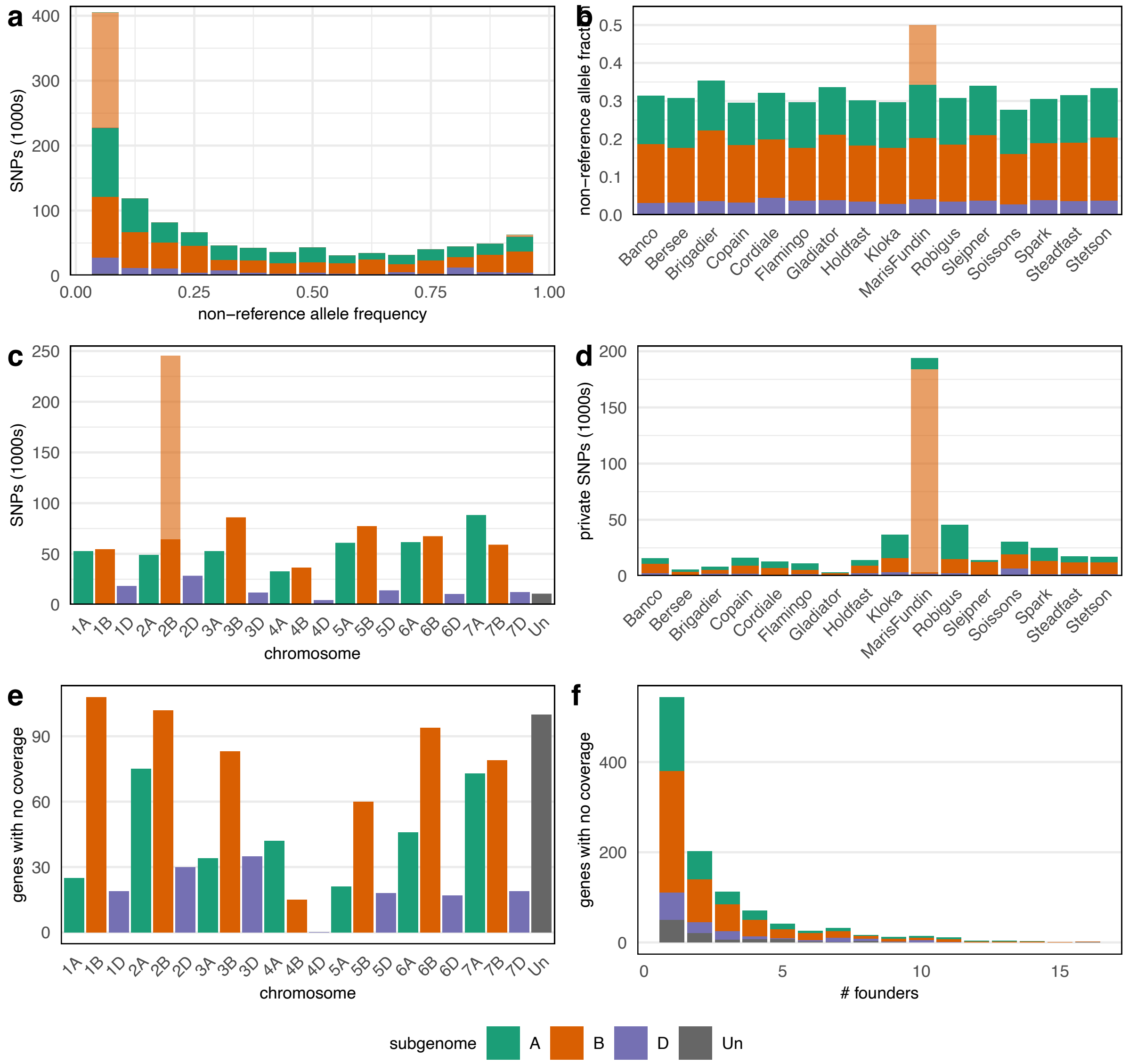

Fig. S1: Distribution of SNPs across chromosomes and founders. (a) shows the non-reference allele frequency spectrum, (b) shows the proportion of non-reference alleles called in each founder, (c) shows the chromosomal distribution of SNPs and (d) shows the distribution of private SNPs (minor allele found in only one founder) across founders. There are a large number of SNPs that are private to Maris Fundin and found on chromosome 2B. There are likely due to an introgression from T. timopheevi and are indicated using a paler shade in a-d. (e) shows the fraction of gene-promoter pairs that have no coverage in at least one founder by chromosome. (f) shows the number of founders that are missing alignments for particular gene-promoter pairs. Colours indicate subgenome locations throughout, with fewer SNPs segregating on the D subgenome, which was acquired in the most recent allo-polyploidisation event.

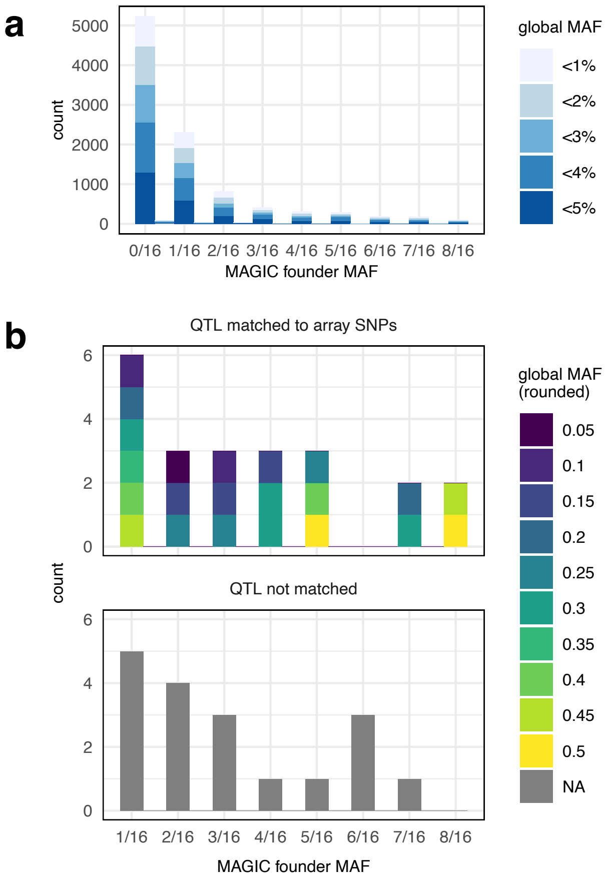

Fig. S2 Minor Allele Frequency (MAF) among ~4,500 global wheats (global MAF) and the NDM founders (MAGIC founder MAF). (a) shows the frequency of 10,111 globally rare variants (global MAF < 5%) among the NDM founders. (b) shows the MAGIC founder MAF of 40 QTLs identified using SNP-based association mapping. Of these, 22 had at least one plausible 'match' among the 55k genotyping array SNPs called in global wheats, the global MAF of these SNPs is shown using the colour scale.

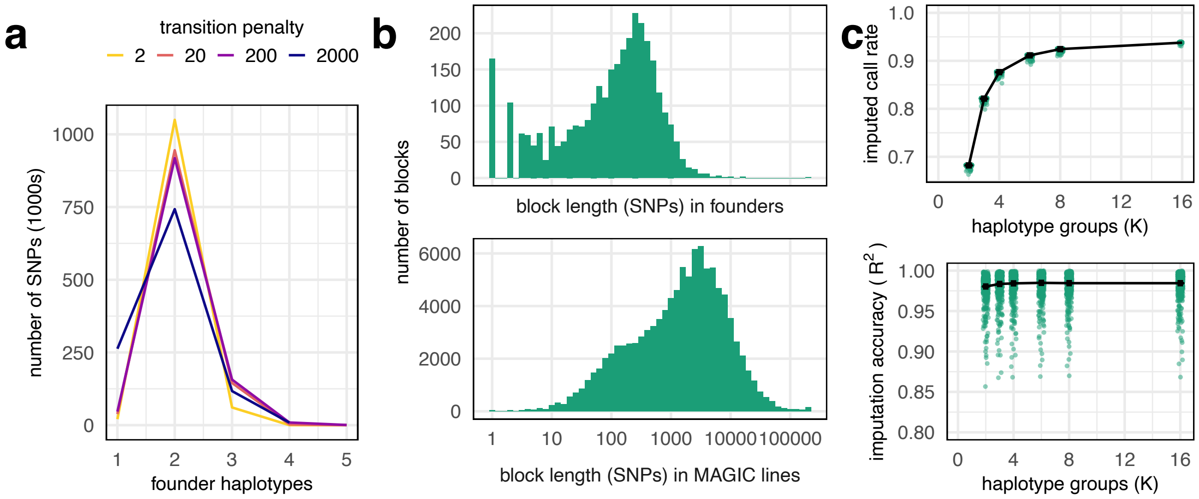

Fig. S3: Haplotype block length in founders and MAGIC lines. Founder haplotype blocks were determined using a dynamic programming algorithm that compares the similarity between all pairs of founders. (a) For various transition penalty parameters, two haplotypes are inferred across most of the genome. In the main text, we refer to the transition penalty 200. (b) The block lengths (in SNP numbers) in the founders are smaller than those of the MAGIC lines. In MAGIC lines, haplotype blocks are defined as consecutive SNPs that are inferred to have the same founder ancestry (>90% dosage). (c) Imputation call rate and accuracy when assuming that MAGIC line mosaics are defined by a different numbers of haplotype groups.

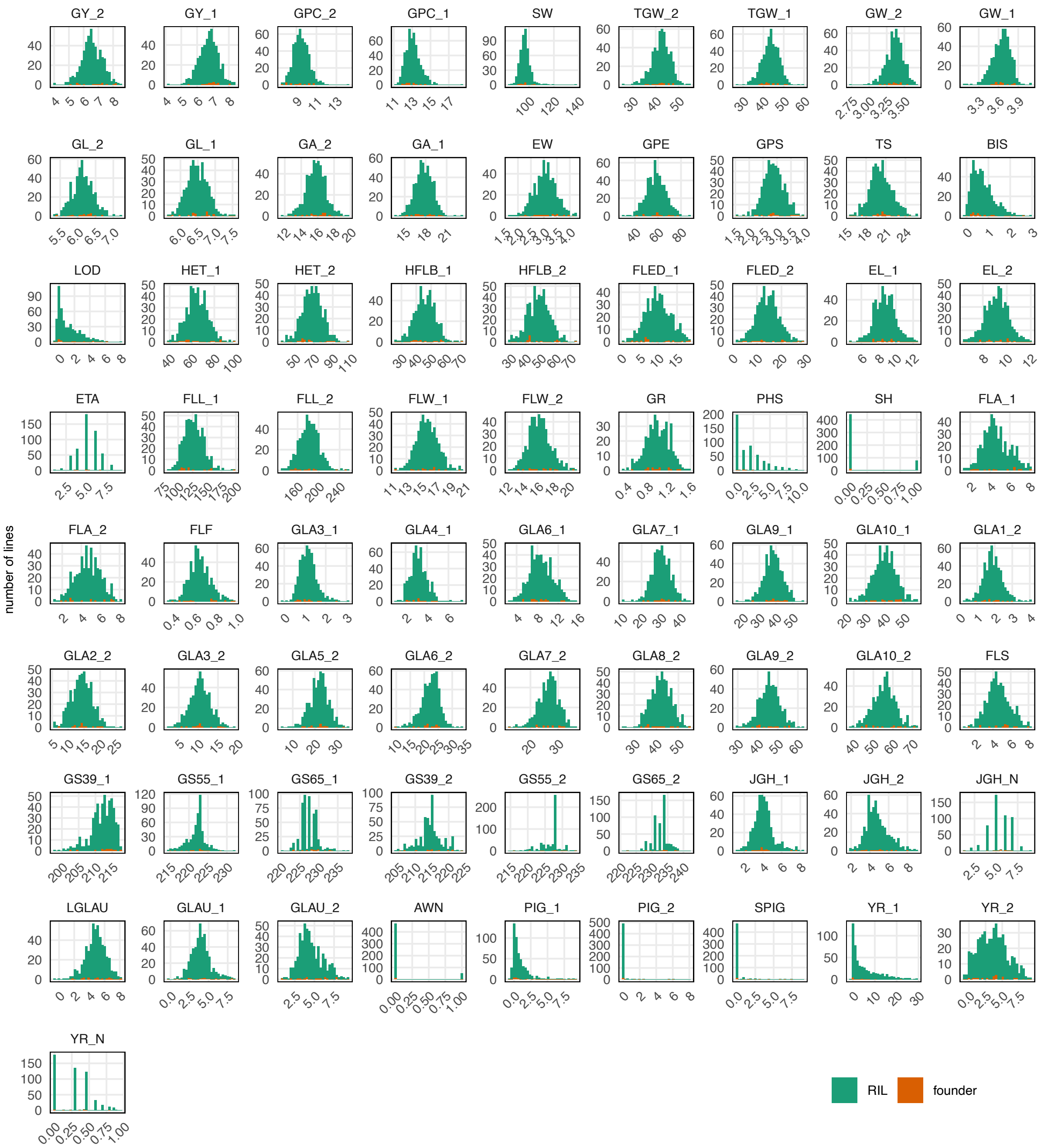

Fig. S4 Distribution of Best Linear Unbiased Estimates (BLUEs) for founders (orange) and the RILs derived from them (green) across 73 phenotypic measurements. For most normally-distributed phenotypes, the phenotypic range of the RILs is wider than that of the founders (transgressive segregation).

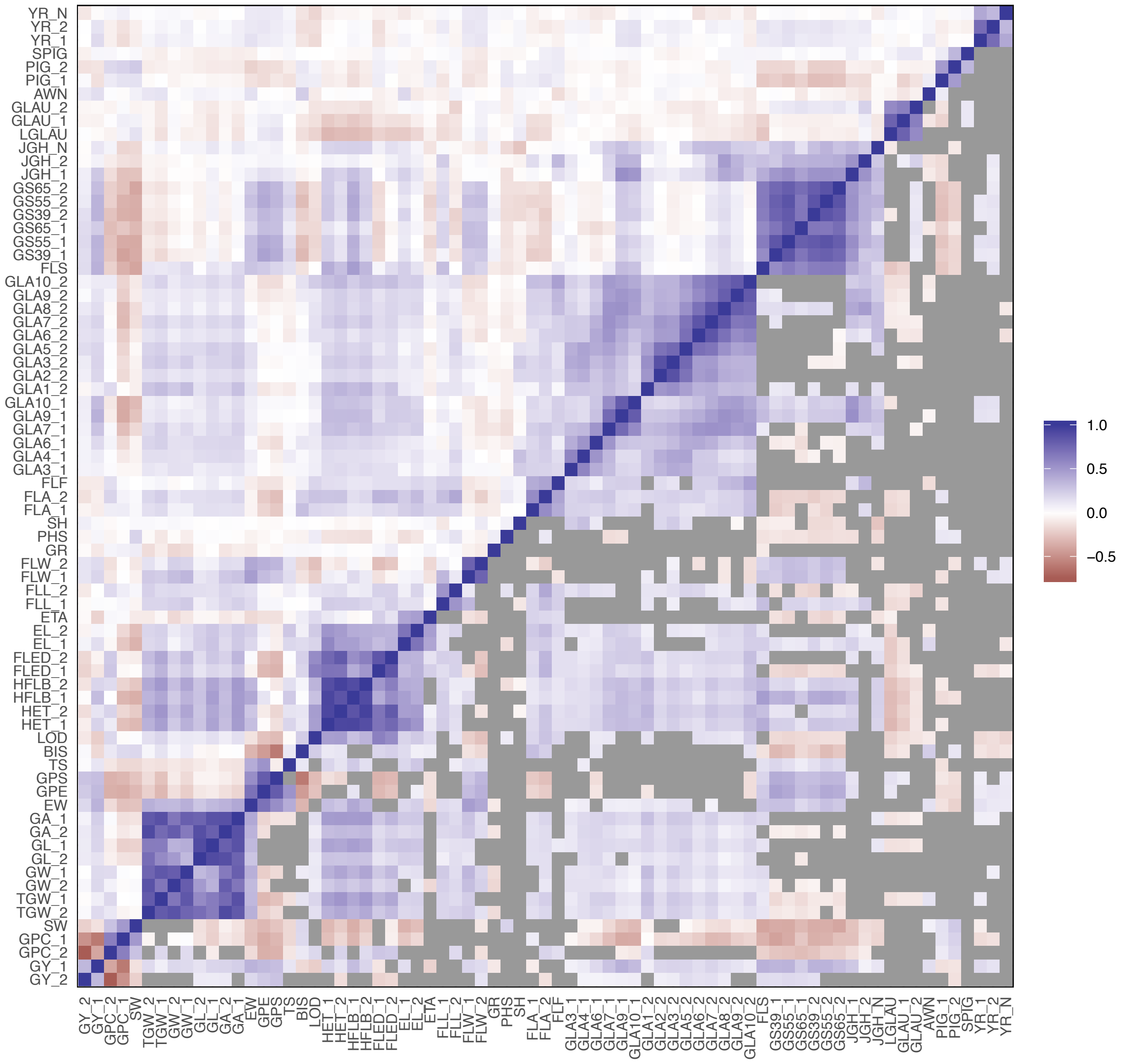

Fig. S5 Correlations between phenotypes in the 504 RILs. The heatmap fill colour shows the Pearson's correlation coefficient for all pairwise combinations of phenotype measurements. In the lower diagonal, all non-significant correlations (p>0.05 for the null hypothesis of zero correlation) are grey.

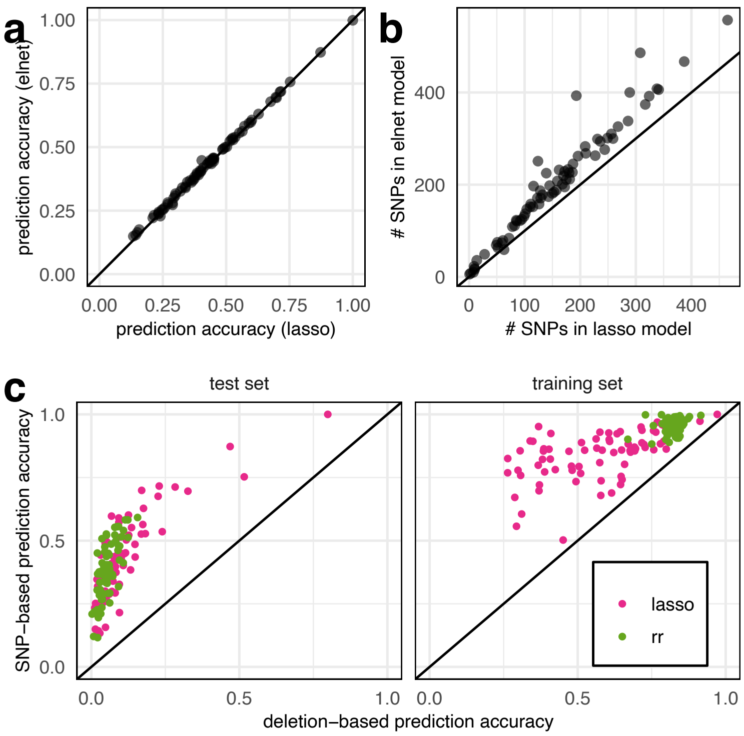

Fig. S6 Comparison between genomic prediction using LASSO and ELNET (a,b) and between genomic prediction based on SNPs and gene deletion scores (c). SNP-based prediction accuracy using LASSO and ELNET are almost identical (a), based on the mean correlation coefficient between predictions and phenotypes for the test set of lines across 50 cross-validation replicates. However, the LASSO prediction models include far fewer SNPs (b), where we show the number of SNPs included in the full model trained on all 504 RILs. (c) shows that the prediction accuracy of models using gene deletion scores never exceeds that of SNP-based prediction.

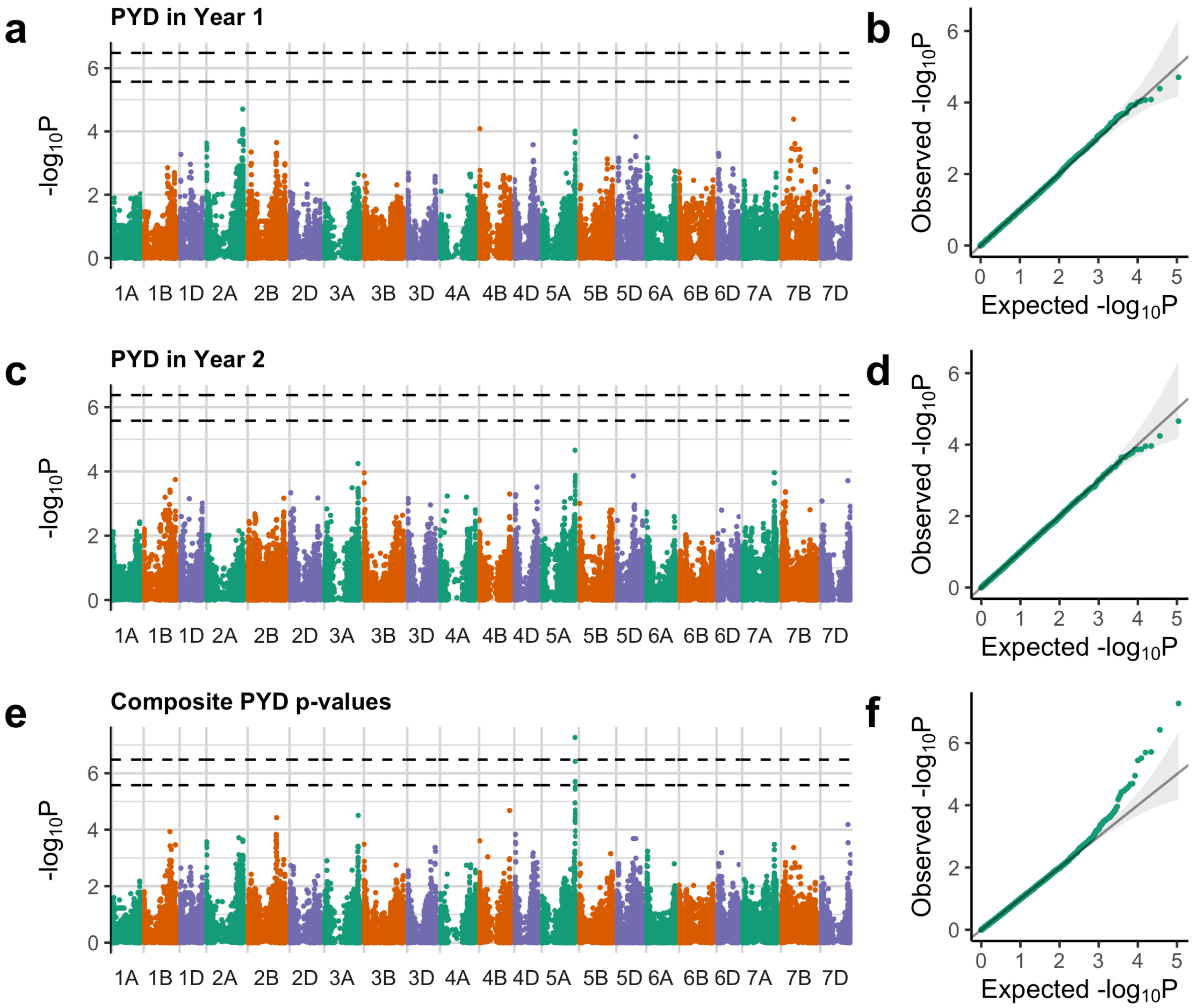

Fig. S7 Association mapping of the Protein Yield Deviation (PYD) compound phenotype. (a) and (c) show Manhattan plots for SNP-based association mapping on the PYD phenotype measured in year 1 and year 2, respectively. (b) and (d) show qq-plots of the p values from these association tests. (e) and (f) show the SNP associations and qq-plot obtained by combining p values between years 1 and 2 using Fisher's method. The locus on chromosome 5A containing a SNP that fully predicts the presence of awns is genome-wide significant.
