# Supplementary material for: Limited haplotype diversity underlies polygenic trait architecture across 70 years of wheat breeding
Source: Genome Biol. 2021 May 6;22:137. doi: 10.1186/s13059-021-02354-7 (PMC8101041; doi:10.1186/s13059-021-02354-7)
Supplement: Supplementary file 3 — Additional file 3. Review history. [file 13059_2021_2354_MOESM3_ESM.docx]

Review History

**First round of review**

**Reviewer 1**

**Are you able to assess all statistics in the manuscript, including the appropriateness of statistical tests used?** Yes, and I have assessed the statistics in my report.

**Comments to author:**

The manuscript presents genomic and phenomic analyses of MAGIC population created by crossing 16 founder lines selected to represent the genetic diversity of large wheat collection. The authors used targeted (both exome and promoter regions) and whole genome sequencing strategies to characterize SNP diversity and use this information for imputing genotypes in the MAGIC population that was characterized by Skim-seq. This part of the manuscript provides quite valuable information for the wheat community on the usage of Skim-seq method for genome imputation using densely genotyped founder lines. It is great to see that at relatively shallow genome coverage (0.07x) it is possible to achieve high accuracy of genotype imputation. The authors also report the first application of the wheat regulatory capture assay for characterizing genetic diversity in wheat and studying the genetics of complex traits.

It is quite interesting to see that the haplotypic diversity of most of the genic regions in such diverse population of founders was relatively low. Not sure whether it is common trend for wheat, or it is caused by selection of founder lines, but it suggests that by expanding our sequencing efforts to additional lines we will unlikely substantially increase the number of discovered haplotypes. I would love to see that the authors to comment on this issue in their discussion. In this context, I think that one additional piece of analysis to be added is more detailed characterization of rare haplotypes, how many are captured by MAGIC population and what is their role in phenotypic variation (for specific comments see below).  
It is exciting to see a detailed analysis of phenotypic variation using such high-density SNP dataset and focus on trade-off between yield and protein content traits. This part of the analysis provides some information that might be useful for breaking negative trade-off. As I pointed out in my comments below, I feel that this section of manuscript would benefit from adding more detailed description of results.

Overall, this is an excellent example how combination of genomics, large scale phenotyping and genetic resources could be used for dissecting trait variation in crop with complex allopolyploid genome.

Below I have few comments that I hope will help to improve the manuscript.
 

L. 131: It would be useful to estimate how many rare alleles from the global population (say with MAF < 0.01) segregate in NDM at high frequency. This provides an opportunity to assess their effects on trait variation, a task otherwise impossible to do in diverse populations due reduced power or fixation of common allele in both parents of bi-parental populations.  

L. 272-287: It would be very interesting to link these QTL mapping summaries with information about the frequency of trait-associated alleles in global populations. What is the relative contribution of rare and common alleles to trait variation in NDM?

L. 330: I believe that this is quite strong statement and it is hard on provided evidence to exclude gene deletion as possible causal factor. It is possible that regions carrying gene deletions are in strong LD with distinct set of SNPs uniquely tagging these regions. As a result, it is difficult to separate the effects of SNPs and gene deletions on trait variation.  

Minor comments:
L. 272: Please provide LD threshold for pruning and to which population this threshold was applied: founding lines or RILs.

L. 289: Possible typo. There should be reference to Fig. 4c.

L. 447: Would it be possible to provide more information about PYD analysis and may be even provide data on genetic mapping of alleles contributing to PYD?

**Reviewer 2**

**Are you able to assess all statistics in the manuscript, including the appropriateness of statistical tests used?** No, I do not feel adequately qualified to assess the statistics.

**Comments to author:**

Scott et al. report the creation and genotyping of a wheat MAGIC population with 16 parents. They also map a large number of traits based on two years of field data. Finally, the authors perform genomic prediction using a variety of approaches.

There is a lot to like about the manuscript, and I had a generally favorable impression. That said, there are several portions of the manuscript that would benefit from improved analysis or more careful exposition.

Major changes:

1) The analysis of haplotype diversity is a headline result but is somewhat confusing both in terms of formulation and presentation. First, line 151 that introduces the analysis starts off talking about 1.13 million "sites." This is a relatively indirect approach for the introduction of the number of discovered nucleotide variants. As presented, the counting of haplotypes at a locus appears to involve an ad hoc approach based on a "dynamic programming algorithm." The approach appears to be explained in much greater detail in the Davies et al. paper cited here. At least a portion of that explanation should carry into the present manuscript. The splitting of "haplotypes" at diverse loci, as explained around line 163, will tend to minimize the number of loci with haplotypic diversity. It should be clear what constitutes a unique haplotype segment in this analysis and how others could reproduce this analysis. As mentioned around line 168 regarding similar analysis in Arabidopsis, the number of unique haplotypes analysis could also focus on the number of potentially functional variants, including amino acid changes, premature stop codons, etc. In short, the haplotype diversity analysis would benefit from a quick Variant Effect Predictor (VeP) run that looks at how much functional variation is likely present at a typical wheat locus. How many unique amino acid combinations are found at each locus (or haplotype as defined here)? Also, the discussion of haplotype frequency would benefit for a clearer presentation (around line 152), perhaps by discussion of the haplotype frequency spectrum (see https://doi.org/10.1534/genetics.104.032219).

The limited number of haplotypes is mention in the Abstract, but the expected number of observed haplotypes is a function of nucleotide diversity, recombination rate, sample size, locus length. Are haplotype numbers lower than expected under a neutral coalescent?

2) MAGIC populations have the advantage that phenotypes can be measured in both founders (initial parents) and progeny; see https://doi.org/10.1534/genetics.106.069641. The authors hint at comparisons or founders and individual RILs (e.g., line 304), but the distribution of trait values relative to the founders is never explicitly explored. Does the distribution phenotypic variance in the RILs fall within the distribution observed with the parents, or does the phenotypic variance exceed that in the parents in any instances?

3) The code used for read mapping, comparative population analyses, and genomic prediction analysis should all be included in a CVS (concurrent versions system) repository for the manuscript. The authors note that the Github repository is "awaiting URL." Github URLs are author-generated. It would also be helpful to include the annotated VCF for individual samples. These files are large and would likely need to go to Figshare or a similar repository. Makin VCFs and annotations available dramatically improves the reproducibility of the research, as SNP calling is difficult to reproduce, even with all source files available.

Minor changes

⁃	Line 106 - Selecting a diverse set of accessions base based on marker data can violate random sampling assumptions of analytical approaches because it can produce an overdispersed sample.

In summary, this is a good manuscript that could be improved by clarification of portions of the analysis and also by making more of the code used to generate results available.

**Authors Response**

**Point-by-point responses to the reviewers’ comments:**

*Reviewer #1: The manuscript presents genomic and phenomic analyses of MAGIC population created by crossing 16 founder lines selected to represent the genetic diversity of large wheat collection. The authors used targeted (both exome and promoter regions) and whole genome sequencing strategies to characterize SNP diversity and use this information for imputing genotypes in the MAGIC population that was characterized by Skim-seq. This part of the manuscript provides quite valuable information for the wheat community on the usage of Skim-seq method for genome imputation using densely genotyped founder lines. It is great to see that at relatively shallow genome coverage (0.07x) it is possible to achieve high accuracy of genotype imputation. The authors also report the first application of the wheat regulatory capture assay for characterizing genetic diversity in wheat and studying the genetics of complex traits.*

We thank the reviewer for their thoughtful and positive overview of our analysis. Since the reviewer highlighted this as a point of interest, we expand on the usefulness of including regulatory sequence – especially in the light of recent analyses on the wheat pan-genome (Response 1.1).

*It is quite interesting to see that the haplotypic diversity of most of the genic regions in such diverse population of founders was relatively low. Not sure whether it is common trend for wheat, or it is caused by selection of founder lines, but it suggests that by expanding our sequencing efforts to additional lines we will unlikely substantially increase the number of discovered haplotypes. I would love to see that the authors to comment on this issue in their discussion.*

We have expanded this part of the discussion as suggested (Response 1.2.1). The founders were chosen to capture genetic diversity from a panel of UK varieties released since 1935. We now state that we therefore expect diminishing numbers of unsampled haplotypes from further sequencing within this germplasm.

Since the last submission, very small sequence differences between assemblies (<99.95% similar) have been used to define haplotypes in 15 diverse wheats of global origin (https://doi.org/10.1038/s42003-020-01413-2). This analysis suggests more haplotypes segregate globally but the method is also likely to be very sensitive such that the haplotypes probably reflect very recent relatedness. For example, the bottleneck that reduces diversity on the D subgenome has no impact on the number of haplotypes per locus using the assemblies alignment method, whereas SNP diversity is hugely reduced on the D subgenome; we find approximately 50% of genes have no SNP diversity on the D subgenome (see also Supplementary Figure 1).

The most appropriate threshold for haplotypic similarity and block size will depend on the research question.. In this case, we are mainly interested in breeding applications and effects on phenotypic variation. We now emphasise the fact that association mapping using founder-ancestry is able to distinguish haplotypic/multi-allelic effects from bi-allelic effects (captured by SNPs) on phenotypic variation. However, the QTLs we mapped are generally functionally biallelic (Response 1.2.2 and Response 1.2.3). We have also carefully described the different definitions of haplotype (and their uses) in the Results (Response 1.2.4).

*In this context, I think that one additional piece of analysis to be added is more detailed characterization of rare haplotypes, how many are captured by MAGIC population and what is their role in phenotypic variation (for specific comments see below).*

To address this point, we used the set of ~55k genotyping array SNPs called in all 16 founders and in 4,500 traditional and modern global wheats. In Supplementary Figure 2, we now show a histogram of variants in this set that are globally rare (<5%). Of these almost half (48%) segregate at some frequency among the MAGIC founders. Most globally rare variants are present in one founder, but some are found at higher frequencies, as displayed in Supplementary Figure 2. Thus, a reasonable fraction of globally rare genetic variation is segregating in the population. However, this analysis is limited by the comparison with genotyping array data, which may be biased against very rare variants. We discuss the potential phenotypic consequences of rare variation further in response to the specific comments below. See Response 1.3.

*It is exciting to see a detailed analysis of phenotypic variation using such high-density SNP dataset and focus on trade-off between yield and protein content traits. This part of the analysis provides some information that might be useful for breaking negative trade-off. As I pointed out in my comments below, I feel that this section of manuscript would benefit from adding more detailed description of results.*

As suggested, we have expanded the section that describes the trade-off between protein content and yield. In particular, we have clarified the description of the Protein-Yield Deviation (PYD) measurement, adding details. Furthermore, we have added Supplementary Figure 7, which shows tests for genome-wide associations between SNPs and PYD. This figure shows that no loci reach genomewide significance when data from each year is analysed separately. However, when we combine the p-values of association for both years using Fisher’s method, we find that the ALI-1 awns locus exceeds the genomewide significance threshold. Supplementary Figure 7 shows the distribution of PYD values for lines with and without awns, where lines with awns tend to have higher PYD values. Thus, if replicated in other populations, this could be an interesting locus and phenotype for selection towards breaking this negative trade-off. The heritability and genomic prediction analyses also suggest some other segregating genetic variation, with smaller effects, that may break this trade-off. See Response 1.4

*Overall, this is an excellent example how combination of genomics, large scale phenotyping and genetic resources could be used for dissecting trait variation in crop with complex allopolyploid genome.*

*Below I have few comments that I hope will help to improve the manuscript.*

*L. 131: It would be useful to estimate how many rare alleles from the global population (say with MAF < 0.01) segregate in NDM at high frequency. This provides an opportunity to assess their effects on trait variation, a task otherwise impossible to do in diverse populations due reduced power or fixation of common allele in both parents of bi-parental populations.*

As discussed above, we have added Supplementary Figure 2, which shows the frequency of variants that are globally rare using different thresholds (MAF<0.01, MAF<0.02 etc.).

*L. 272-287: It would be very interesting to link these QTL mapping summaries with information about the frequency of trait-associated alleles in global populations. What is the relative contribution of rare and common alleles to trait variation in NDM?*

As pointed out by the reviewer, variants that segregate in our population can potentially be associated with the measured phenotypes. We attempted to relate the global SNP frequencies to the genomewide significant QTLs that we identified. We looked in the ~55k SNPs called in the global wheats for those that were within the mapping intervals for the 40 QTLs identified in our SNP-based mapping. These overlapping sites were then filtered to include only those that had NDM founder genotype calls that were consistent with the peak QTL association. 22/40 QTL could be ‘matched’ in this way. We note that these matches can’t be given high confidence because the LD between these SNPs and the underlying causal variation could break down in the global population. Based on these 22 ‘matched QTLs’, Supplementary Figure 2b shows the allele frequencies of the QTLs in the global population.

Focussing on rare causal variation, the two rarest that could be matched are:

(1) A Yellow Rust QTL (global MAF 6.7%), this is a known locus and is an introgression from A. ventricosa.

(2) A QTL for Grain Yield in Year 2 on chromosome 3D between 11.5mb and 23.8mb (global MAF 10.4%), this is a marginal (significant at 5% genomewide threshold but not 1%) but possibly interesting locus that we weren’t able to colocalise. This locus explains 5% of the phenotypic variance in the population, and the positive allele is carried by Banco, MarisFundin, and Soissons.

Again, we note that the genotyping array may be biased towards the detection of common variation and we may therefore be underestimating the degree to which rare functional alleles have been detected in our population.

See Response 1.5.

*L. 330: I believe that this is quite strong statement and it is hard on provided evidence to exclude gene deletion as possible causal factor. It is possible that regions carrying gene deletions are in strong LD with distinct set of SNPs uniquely tagging these regions. As a result, it is difficult to separate the effects of SNPs and gene deletions on trait variation.*

We agree with the reviewer. If SNPs were serving as good surrogates for a causal deletion, then the associations should be as strong as each other. We don’t see that: the gene deletion associations often track the SNP associations but are usually weaker. This is the evidence that suggests that deletions they may not be causal. However, our results are also consistent with the quantitative measure of deletion that we use being too noisy. Furthermore, we rely on alignments to the reference genome for Chinese Spring, such that important presence and absence variation may be missed. In summary, we think that while gene deletions might be responsible for some of the QTLs, we have not found overwhelmingly strong evidence supporting the deletion of any particular candidate gene to the exclusion of SNP effects. We now discuss these caveats to our analysis in Response 1.6.

*Minor comments:*

*L. 272: Please provide LD threshold for pruning and to which population this threshold was applied: founding lines or RILs.*

As suggested, we have added the relevant LD pruning information from the methods section (Response 1.7.1) here to improve clarity and readability of the main text (Response 1.7.2).

*L. 289: Possible typo. There should be reference to Fig. 4c.*

Thank you for spotting this accidental incorrect cross-reference. We have changed ‘Figure 4b’ to ‘Figure 4c’ here (Response 1.8).

*L. 447: Would it be possible to provide more information about PYD analysis and may be even provide data on genetic mapping of alleles contributing to PYD?*

As discussed above, we have clarified how the PYD phenotype was calculated in the main text (Response 1.4). We also discussed association mapping for PYD and added Supplementary Figure 7, which reveals a potential association between awns and PYD. We thank the reviewer for expressing an interest in this part of the analysis and we think the overall manuscript is more compelling as a result of increased emphasis on these results.

*Reviewer #2: Scott et al. report the creation and genotyping of a wheat MAGIC population with 16 parents. They also map a large number of traits based on two years of field data. Finally, the authors perform genomic prediction using a variety of approaches.*

*There is a lot to like about the manuscript, and I had a generally favorable impression. That said, there are several portions of the manuscript that would benefit from improved analysis or more careful exposition.*

We thank the reviewer for their positive appraisal and for highlighting points for improvement. We respond in detail below.

*Major changes:*

*1) The analysis of haplotype diversity is a headline result but is somewhat confusing both in terms of formulation and presentation. First, line 151 that introduces the analysis starts off talking about 1.13 million "sites." This is a relatively indirect approach for the introduction of the number of discovered nucleotide variants.*

We thank the reviewer for pointing out the lack of clarity in this passage, which has been re-written. We have added a new subsection heading to our description of haplotype diversity among founders. For clarity, we introduce this section of the results by discussing usages of the term ‘haplotype’ in crop genetics (Response 1.2.4). In our study, we discuss the haplotypic diversity among the founders, e.g., number of distinct SNP combinations found among founders at each gene, and we also discuss larger ‘founder haplotypes’, which are genomic blocks in MAGIC RILs inherited from particular founders. We believe these two distinct and common usages of the term haplotype were confusing and hope that the revision makes this much clearer. We give further details about how our description of this analysis has been revised below.

*As presented, the counting of haplotypes at a locus appears to involve an ad hoc approach based on a "dynamic programming algorithm." The approach appears to be explained in much greater detail in the Davies et al. paper cited here. At least a portion of that explanation should carry into the present manuscript. The splitting of "haplotypes" at diverse loci, as explained around line 163, will tend to minimize the number of loci with haplotypic diversity. It should be clear what constitutes a unique haplotype segment in this analysis and how others could reproduce this analysis.*

This passage has been expanded and re-phrased to make it clearer (Response 2.1.1). We now clarify across two paragraphs that we used two methods to characterise haplotype diversity.

First, we used a single-link clustering of SNPs within gene-promoter pairs as implemented in R (also described in the methods, Reponse 2.1.2). The results from this clustering analysis are shown in Figure 2b. A limitation of this gene-centric analysis is that it doesn’t look for evidence that haplotypes extend beyond single genes

Therefore, we also used a dynamic programming algorithm that dynamically infers where the haplotype block boundaries are by categorising founder pairs as being either similar or different at each locus. Results from this analysis are show in Figure 2c and Supplementary Figure 3. We have expanded the description of the method in the results; it is also described in the methods (Response 2.1.3).

The sentence about splitting haplotypes (formerly on Line 163, now at the end of Response 2.1.1) is to explain the comparison between the two methods. We believe the re-writing of this sentence and surrounding text has made this clear.

To replicate the dynamic programming algorithm, we provide an R function to perform this analysis on GitHub. We also provide the founder genotype data and R scripts to produce the plots on the project website (http://mtweb.cs.ucl.ac.uk/mus/www/MAGICdiverse/index.html), where we are able to host the data together with the script for simple replication.

This dynamic programming method is not from the Davies et al. STITCH paper, which was the software used for imputation rather than haplotype inference. However, our previous presentation was not clear because the haplotypic diversity among founders was mentioned again in the section about imputation. We have now clarified this issue throughout the manuscript.

*As mentioned around line 168 regarding similar analysis in Arabidopsis, the number of unique haplotypes analysis could also focus on the number of potentially functional variants, including amino acid changes, premature stop codons, etc. In short, the haplotype diversity analysis would benefit from a quick Variant Effect Predictor (VeP) run that looks at how much functional variation is likely present at a typical wheat locus. How many unique amino acid combinations are found at each locus (or haplotype as defined here)?*

As suggested, we have used Variant Effect Predictor to examine the predicted functional effects of called SNP variation. The results are summarised in Response 2.2.1. Of the 1.13M SNPs called in genic and promoter regions, ~95k were ‘missense variants’ predicted to directly affect amino acid sequence. That is, our SNP set contains roughly one SNP per gene that affects amino acid sequence. Because this marker density is insufficient for per-gene clustering, we didn’t repeat the gene-centric haplotype analysis using only this SNP set. We note that these results are compatible with those from a recent analysis of 15 wheat genome assemblies, which find that non-coding flanking sequences are required to distinguish haplotypes in wheat (https://doi.org/10.1038/s42003-020-01413-2).

We note that we are likely to underestimate the true extent of SNP variation that affects amino acid sequence due to the filtering steps. We applied standard filters for SNP detection and genotyping in polyploid wheat (Response 2.2.2), e.g., https://doi.org/10.1093/gigascience/giz018, which describes the genic/promoter capture probes we used). First, it will be difficult to get confident read alignments to genes that have highly similar homeologues on the other subgenomes and these alignments may be removed by mapping quality filters. To some extent, this problem is mitigated when using 2x150bp reads. Second, misalignment of reads from other homeologues can appear as heterozygous calls. Therefore, we removed all sites where any founder had a heterozygous call. Third, the reference genome for Chinese Spring may have important structural differences from the sequenced varieties. For example, genome assembly and re-annotation of *Arabidopsis thaliana* MAGIC population founders revealed that a simple lift over of variants would incorrectly predict functional effects due to exon rearrangements such as changes in splicing (https://doi.org/10.1038/nature10414). Finally, we did not consider indel variation. Thus, our SNP set is more appropriately used as a dense set of tagging genotypes, rather than a complete functional characterisation of the founders. Nevertheless, the sequence data and alignments might serve as a resource for more detailed functional investigations in the future.

We believe the fundamental motivation behind this suggestion is to determine the functional relevance of haplotypic diversity. The revised description of the analysis of haplotypes among founders and MAGIC haplotype mosaic construction now makes it clear that we can distinguish all the founders in the MAGIC haplotype mosaics. Therefore, we now point out that the founder-haplotype-based QTL mapping would reveal any genomewide significant effects on trait variation that are multi-allelic, even if the underlying causal variants are not observed directly. We do not much find evidence for multi-allelic QTLs: 2/42 QTLs are detectable using founder-haplotype-based mapping but not SNP-based mapping. This suggests that most effects on trait variation are biallelic. This suggests our finding that most genes have no more than 2 haplotypes at most loci is both true and relevant for breeding (Response 1.2.2 and Response 1.2.3).

*Also, the discussion of haplotype frequency would benefit for a clearer presentation (around line 152), perhaps by discussion of the haplotype frequency spectrum (see https://doi.org/10.1534/genetics.104.032219).*

*The limited number of haplotypes is mention in the Abstract, but the expected number of observed haplotypes is a function of nucleotide diversity, recombination rate, sample size, locus length. Are haplotype numbers lower than expected under a neutral coalescent?*

As discussed above, we have clarified the section on haplotype frequencies. We plot the haplotype frequency spectrum here.

Figure 1 Spectrum of haplotype frequencies in the 16 founders. The x-axis shows the 73,982 gene-promoter pairs that have called SNP variation. These are sorted first by the number of haplotypes, then by the frequency of the most common haplotypes, in turn. The colours show the different haplotypes called at each gene-promoter pair using single link clustering with a 95% similarity threshold. The y-axis shows the frequency of these haplotypes among the 16 founders.

The referenced paper uses the full haplotype frequency distribution to test for neutrality. As the reviewer points out, it would also be interesting to test for selection/neutrality across the genome in wheat and to infer other relevant population genetic properties such as the effective population size. However, we are limited in our ability to perform such tests. First, the founders are not randomly sampled, as noted below. Second, the founders span ~70 years, which makes the relevant coalescent process non-standard. Third, these varieties are related through specific pedigrees rather than randomly mating populations of the type envisioned in standard coalescent models. Thus, ‘gene drop’ tests for neutrality using large pedigrees might be more appropriate in wheat (https://doi.org/10.1371/journal.pbio.3000071). Finally, 16 samples is likely to be insufficient for analytical tests of selection or demography.

Instead, we focus on cumulative haplotype counts and their functional consequences, as discussed above. These counts are then compared to some comparable analyses in wheat and Arabidopsis (Response 1.2.1 and Response 1.2.2). For this reason, we believe the haplotype summaries given in Figure 2b and Supplementary Figure 3a are clearer presentations of the data.

*2) MAGIC populations have the advantage that phenotypes can be measured in both founders (initial parents) and progeny; see https://doi.org/10.1534/genetics.106.069641. The authors hint at comparisons or founders and individual RILs (e.g., line 304), but the distribution of trait values relative to the founders is never explicitly explored. Does the distribution phenotypic variance in the RILs fall within the distribution observed with the parents, or does the phenotypic variance exceed that in the parents in any instances?*

The phenotype distributions for the RILs and founders are shown in Supplementary Figure 4. We also summarise these distributions with particular reference to whether the extreme phenotypes observed in the RILs is more extreme than that of the founders (transgressive segregation). We have expanded this section to discuss transgressive segregation for a selected trait, yield, which has greater negative than positive transgressive segregation due to selection on the founders that wasn’t applied to the RILs (Response 2.3). For all QTLs, we give the distribution of estimated founder haplotype effects and the founders that carry the peak SNP in Supplementary Table 7.

*3) The code used for read mapping, comparative population analyses, and genomic prediction analysis should all be included in a CVS (concurrent versions system) repository for the manuscript. The authors note that the Github repository is "awaiting URL." Github URLs are author-generated. It would also be helpful to include the annotated VCF for individual samples. These files are large and would likely need to go to Figshare or a similar repository. Makin VCFs and annotations available dramatically improves the reproducibility of the research, as SNP calling is difficult to reproduce, even with all source files available.*

We apologise that the appropriate links were not accessible. These should have appeared in the submitted manuscript under ‘availability of data and materials’ (Response 2.4), unless this section is not sent for review.

The GitHub address is <https://github.com/michaelfscott/DIVERSE_MAGIC_WHEAT> . Alongside the relevant custom scripts (e.g., mixed model association mapping and founder haplotype comparison), this github repository now also hosts the scripts used for alignment, variant calling, imputation, and genomic prediction, as suggested.

We provide VCFs from the European Variant Archive (EVA) for the SNP variants called in the founders (EVA accession ERZ1643321). At these SNP sites, we then made genotype calls in the inbred lines (i) by using the alignments to make genotype calls directly (EVA accession ERZ1643320) and (ii) by imputation (EVA accession ERZ1643322).

We also provide further data on <http://mtweb.cs.ucl.ac.uk/mus/www/MAGICdiverse/index.html>, e.g., phenotypes and founder/RIL genotypes in PLINK format. We provide R functions to replicate the haplotype comparison between founders (dynamic programming algorithm) and perform genetic mapping using SNPs and/or founder haplotypes. This project website allows us to host R scripts to run the core analysis pipelines (e.g., genetic mapping), which can be downloaded alongside the relevant data to provide a simple way of replicating our analysis. We hope these scripts will be useful for researchers interested in collecting new phenotypes in the population, which can then be easily mapped using the exisiting genetic data.

*Minor changes*

*⁃ Line 106 - Selecting a diverse set of accessions base based on marker data can violate random sampling assumptions of analytical approaches because it can produce an overdispersed sample.*

This is correct and further argues against using tests to infer selection/neutrality using the haplotype frequency spectrum among founders (or similar population genetic analyses). We don’t perform any analyses that assume the founders were randomly sampled from a population of interest.

*In summary, this is a good manuscript that could be improved by clarification of portions of the analysis and also by making more of the code used to generate results available.*

We thank the reviewer for highlighting sections of our presentation that were unclear. The revised manuscript has been greatly improved as a result. We have designed this population to be a useful community resource and have therefore aimed for high standards of data/code availability.
